# Auxin-producing bacteria promote barley rhizosheath formation

**DOI:** 10.1038/s41467-023-40916-4

**Published:** 2023-09-19

**Authors:** Feiyun Xu, Hanpeng Liao, Jinyong Yang, Yingjiao Zhang, Peng Yu, Yiying Cao, Ju Fang, Shu Chen, Liang Li, Leyun Sun, Chongxuan Du, Ke Wang, Xiaolin Dang, Zhiwei Feng, Yifan Cao, Ying Li, Jianhua Zhang, Weifeng Xu

**Affiliations:** 1 Center for Plant Water-use and Nutrition Regulation and College of Resources and Environment, Joint International Research Laboratory of Water and Nutrient in Crop and College of JunCao Science and Ecology, Fujian Agriculture and Forestry University, Fuzhou, 350002 China; 2 Fujian Provincial Key Laboratory of Soil Environmental Health and Regulation, College of Resources and Environment, Fujian Agriculture and Forestry University, Fuzhou, 350002 China; 3 Institute of Oceanography, Minjiang University, Fuzhou, 350108 China; 4 Crop Functional Genomics, Institute of Crop Science and Resource Conservation, University of Bonn, Bonn, Germany; 5 Emmy Noether Group Root Functional Biology, Institute of Crop Science and Resource Conservation, University of Bonn, Bonn, Germany; 6 College of Agriculture, Yangzhou University, Yangzhou, 225000 China; 7 State Key Laboratory of Agrobiotechnology, The Chinese University of Hong Kong, Hong Kong, China

**Keywords:** Microbial ecology, Symbiosis, Soil microbiology, Plant symbiosis

## Abstract

The rhizosheath, or the layer of soil closely adhering to roots, can help plants to tolerate drought under moderate soil drying conditions. Rhizosheath formation is the result of poorly understood interactions between root exudates, microbes, and soil conditions. Here, we study the roles played by the soil microbiota in rhizosheath formation in barley (a dry crop). We show that barley rhizosheath formation is greater in acid soil than in alkaline soil, and inoculation with microbiota from acid soil enhances rhizosheath formation in alkaline soil. The rhizosheath-promoting activity is associated with the presence of *Flavobacteriaceae* and *Paenibacillaceae* bacteria that express genes for biosynthesis of indole-3-acetic acid (IAA, a common auxin), as determined by metagenomics and metatranscriptomics. Two bacterial strains isolated from rhizosheath (*Chryseobacterium culicis* and *Paenibacillus polymyxa*) produce IAA and enhance barley rhizosheath formation, while their IAA-defective mutants are unable to promote rhizosheath formation. Co-inoculation with the IAA-producing strains enhances barley grain yield in field experiments through an increase in spike number. Our findings contribute to our understanding of barley rhizosheath formation, and suggest potential strategies for crop improvement.

## Abstract (web summary)

The rhizosheath, or the soil layer closely attached to roots, can help plants tolerate drought. Here, the authors show that rhizosheath formation in barley is promoted by soil bacteria that produce indole-3-acetic acid, a common auxin.

## Introduction

Securing food production under climate change requires an understanding of the critical roles of the rhizosheath in crop water and nutrient use efficiency^1–3^. The rhizosheath is the soil adhering to root systems, which is a consequence of adherence of soil to root hairs and mucilage from roots or microbes^4^. Rhizosheath, as an adaptive trait for desert species, is important for plant performance and acclimation to water deficit^5^. Plants benefit from the rhizosheath by protecting roots from physical impedance^6–8^, enhancing plant production^6^, and increasing water uptake under drought conditions^9–12^. In drying soil, rhizosheath soil is wetter than the surrounding soil, which can decrease soil shrinkage and the probability of air space formation at the root surface^13^. Other nutritional benefits include nutrient uptake^14,15^ and tolerance to scarcity^16,17^. Rhizosheath is a conserved trait in plants^12,18^ and could be leveraged to improve water and nutrient acquisition under drought conditions.

Rhizosheath formation is genetically controlled and environmentally regulated in plants. The positioning and patterning of lateral roots^7,15^ and root hairs^7,8,11,12,17–22^ are essential in rhizosheath formation. For example, the change trends of lateral root number in switchgrass were consistent with rhizosheath weight^7^. Rice, a wet crop, can only form rhizosheath under moderate soil drying (MSD)^10,11,21^. Barley, a dry crop, can form rhizosheath under both well-watered (WW) and MSD conditions^23,24^. Among edaphic factors (soil water content^10,11,17,25^, soil strength^22,26^, and soil reaction^19,20,27,28^) involved in rhizosheath formation, soil pH shows the greatest effect on rhizosheath formation^27^. Soil pH is very important in determining the distributions of specific bacteria and the bacterial community composition^29–32^. However, there is a dearth of studies focused on the roles of microbiota in barley rhizosheath formation in different soil pH (acid or alkaline soil).

Barley (*Hordeum vulgare* L.) is an important cereal crop in terms of quantity produced and area cultivated^33^. Barley, with high drought tolerance, is a model crop for drought research. In this study, we evaluated the roles of the microbiota on rhizosheath formation using wild-type (WT, *Hordeum vulgare* cv. Optic) and its root hair lacking mutant *no root hair* (*nrh*) barley plants^22,24^ in acid or alkaline soil under WW and MSD conditions. We employed 16 S rRNA gene amplicon sequencing of rhizosheath and endosphere in both soil conditions to identify bacteria associated with rhizosheath formation. Further, we conducted metagenomics and metatranscriptomics analyses to investigate the roles of the microbiota on rhizosheath formation in acid or alkaline soil. We then isolated some strains and explored their roles in rhizosheath formation using IAA biosynthesis mutants. Our findings provide some evidences for soil microbiota promoting barley rhizosheath formation under soil drying.

## Results

### Microbiota associated with barley rhizosheath formation

**Fig. 1:**
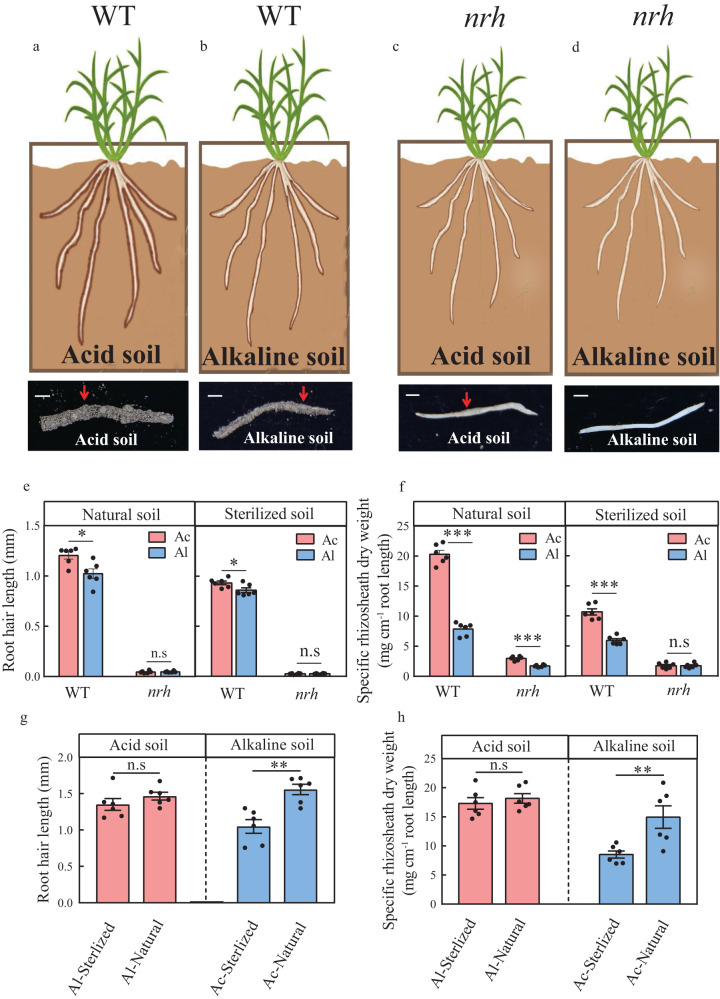
Microbiota is important for rhizosheath formation in acid or alkaline soil under moderate soil drying (with Supplementary Figs. 1, 2 and Supplementary Table 1). **a**–**d** Rhizosheath formation of wild-type (WT) and root hair blocking mutant (*nrh*) barley plants in acid soil (Ac) or alkaline soil (Al) under moderate soil drying (MSD). **e**, **f** Root hair length (**e**) and rhizosheath formation (**f**) of the WT and *nrh* in natural acid or alkaline soil and sterilized acid or alkaline soil (Ac and Al) under MSD. **g**, **h** Root hair length (**g**) and rhizosheath formation (**h**) of barley plants in the presence of sterilized soil slurry (Ac-/Al-Sterilized) or non-sterilized soil slurry (Ac-/Al-Natural) from the rhizosheath of acid or alkaline soil under MSD. Data are means ± SE (*n* = 6 independent replicates). Asterisks indicate a significant difference between soil environments (*p < 0.05, **p < 0.01, ***p < 0.001) by two-sided Student’s *t* test. The exact *p* values are provided in the Source Data file.

To investigate the effect of soil pH on barley rhizosheath formation, acid and alkaline soils were collected from two geographical locations (Fig. 1a–d and Supplementary Table 1). Root hair length and rhizosheath formation in acid or alkaline soils were determined under well-watered (WW) and moderate soil drying (MSD) conditions (Fig. 1e, f and Supplementary Fig. 1a, b). Rhizosheath formation was greater for wild-type plants (WT) than *no root hair* plants (*nrh*), possibly due to the longer root hairs of the WT. Compared to WW conditions, rhizosheath formation in the WT was significantly greater in acid or alkaline soil under MSD (Fig. 1e, f and Supplementary Fig. 1b). No significant difference in root hair length of *nrh* plants was observed between acid soil and alkaline soil under MSD (Fig. 1e). Moreover, a 159% increase in rhizosheath formation was observed in WT grown in acid soil compared to WT grown in alkaline soil under MSD (Fig. 1f).

We then tested whether the rhizosheath microbiota was associated with the barley rhizosheath formation using sterilized soils (Fig. 1e, f). Under MSD conditions, the root hair length of the WT increased approximately 18% in natural acid soil than in natural alkaline soil, whereas the root hair length of the WT was only 9% greater in sterilized acid soil than in sterilized alkaline soil (Fig. 1e). WT plants exhibited 80% greater rhizosheath formation in sterilized acid soil compared to sterilized alkaline soil under MSD (Fig. 1f). Rhizosheath formation of *nrh* plants showed no significant difference between sterilized acid soil and sterilized alkaline soil under MSD (Fig. 1f). Compared to natural soils, rhizosheath formation of WT in sterilized acid soil or sterilized alkaline soil was significantly reduced under MSD, respectively (Fig. 1f). Furthermore, when the rhizosheath microbiota from acid soil was inoculated in alkaline soil, the root hair length and rhizosheath formation of barley plants significantly increased by 48% and 75%, respectively (Fig. 1g, h and Supplementary Fig. 2). However, when the rhizosheath microbiota of alkaline soil was inoculated in acid soil, root hair length and rhizosheath formation increased by only 8% and 5%, respectively, (Fig. 1g, h and Supplementary Fig. 2). These results showed that the microbiota may be associated with barley rhizosheath formation.

### Barley bacterial composition among different rhizocompartments

To elucidate microbial community structures in acid or alkaline soil, we next analyzed the bacterial community diversity and composition in the rhizosheath, endosphere, and bulk soil of WT and *nrh* plants in acid or alkaline soil under WW and MSD using 16 S rRNA gene amplicon sequencing (Supplementary Figs. 3a–d, 4a–e). Chao index in the rhizosheath of WT plants did not differ significantly between acid and alkaline soils under WW or MSD (Supplementary Fig. 3a, 4a). Principal coordinate analysis (PCoA, based on Bray–Curtis distance) revealed that the bacterial communities of the bulk soil and rhizosheath differed significantly between acid and alkaline soils under both WW and MSD conditions (Supplementary Figs. 3b, 4b).

The dominant phyla in the rhizosheath of WT and *nrh* plants included *Proteobacteria*, *Actinobacteria*, *Gemmatimonadota, Firmicutes*, *Bacteroidota*, and *Chloroflexota* under both MSD and WW (Figs. S3c, S4c and Supplementary Data 1a, c). The abundances of *Gemmatimonadota* (Fold Change [FC] = 4.23), *Firmicutes* (FC = 2.79), and *Bacteroidota* (FC = 22.28) in the rhizosheath of WT plants were higher in acid soil compared to alkaline soil under MSD (Supplementary Fig. 4c and Supplementary Data 1c). *Firmicutes* (FC = 2.18) and *Bacteroidota* (FC = 2.51) in *nrh* plants showed a similar trend, with higher abundance in acid soil compared to alkaline soil under MSD (Supplementary Fig. 4c and Supplementary Data 1c). At the family level, *Flavobacteriaceae* (abundance in acid soil under MSD, FC: 7.71%, 96.38), *Paenibacillaceae* (2.22%, 11.68), *Rhodanobacteraceae* (11.36%, 11.59), *Gemmatimonadaceae* (7.07%, 4.23), and *Nitrosomonadaceae* (3.97%, 1.82) in the rhizosheath of WT plants were significantly elevated in acid soil compared to alkaline soil under MSD (Supplementary Fig. 4e and Supplementary Data 1d). The abundances of *Paenibacillaceae* and *Flavobacteriaceae* in the rhizosheath of WT plants were significantly increased in acid soil compared to alkaline soil under WW (Supplementary Fig. 3d and Supplementary Data 1b).

**Fig. 2:**
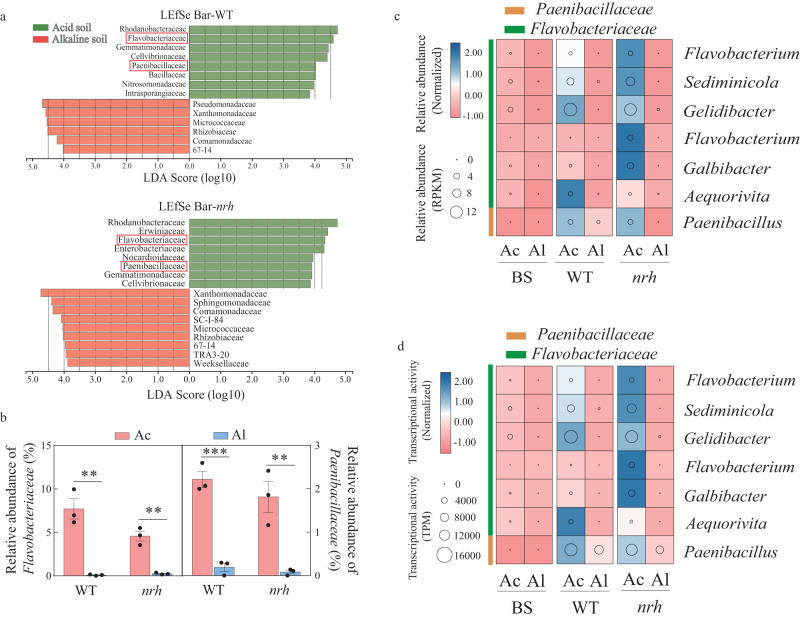
*Flavobacteriaceae* and *Paenibacillaceae* associated with barley rhizosheath formation in acid or alkaline soil under moderate soil drying (with Supplementary Figs. 3–6 and Supplementary Data 1–4). **a** Liner discriminant analysis effect size (LEfSe) analysis of bacterial taxon with significant difference in abundances in WT and *nrh* rhizosheath between acid and alkaline soil. Linear discriminant analysis score ≥3.8. **b** Relative abundances of *Flavobacteriaceae* and *Paenibacillaceae* in WT and *nrh* rhizosheath of acid and alkaline soils. Data are means ± SE (*n* = 3 independent replicates). Asterisks indicate significant differences by two-sided Student’s *t* test (**p < 0.01, ***p < 0.001). **c** Relative abundance of *Flavobacteriaceae* and *Paenibacillaceae* based on MAGs revealed by metagenomics. Circle size represents the relative abundance of each MAG (RPKM). Colors represent the normalized relative abundance. BS bulk soil, Ac acid soil, Al alkaline soil. **d** Transcriptional activity of *Flavobacteriaceae* and *Paenibacillaceae* based on metatranscriptomics. Circle size represents the transcriptional activity values (TPM). Colors represent the normalized relative transcriptional activity. BS bulk soil, Ac acid soil Al alkaline soil. The exact *p* values are provided in the Source Data file.

To investigate the rhizosheath-dependent microbiota of acid and alkaline soils under MSD, linear discriminant analysis (LDA) effect size (LEfSe) analysis was used to evaluate the influence of bacterial biomarkers on rhizosheath formation (Fig. 2a). In the rhizosheath of WT plants, *Flavobacteriaceae* (LDA score, 4.59) and *Paenibacillaceae* (4.03) were specifically enriched in acid soil relative to alkaline soil under MSD (Fig. 2a). In the rhizosheath of *nrh* plants under MSD, *Flavobacteriaceae* (LDA score, 4.33) and *Paenibacillaceae* (LDA score, 3.91) showed similar trends to those in the rhizosheath of WT plants (Fig. 2a). In addition, *Flavobacteriaceae* (7.71%) and *Paenibacillaceae* (2.22%) of the WT rhizosheath in acid soil had significantly higher relative abundances than in alkaline soil (*Flavobacteriaceae*, 0.08%; *Paenibacillaceae*, 0.19%; Fig. 2b and Supplementary Data 1d) under MSD. In the *nrh* plants, *Flavobacteriaceae* (4.57%) and *Paenibacillaceae* (1.81%) was also significantly enriched in acid soil (in alkaline soil: *Flavobacteriaceae* 0.24%; *Paenibacillaceae* 0.08%; Fig. 2b and Supplementary Data 1d).

To further investigate which members of the *Flavobacteriaceae* and *Paenibacillaceae* play roles in rhizosheath formation, we reconstructed prokaryotic metagenome-assembled genomes (MAGs) from rhizosheath in acid and alkaline soils through binning of shotgun metagenomic contigs (Supplementary Fig. 5). A total of 124 non-redundant medium to high quality (estimated completeness ≥50% and contamination ≤5%) MAGs were obtained, including 122 bacteria and 2 archaea (Supplementary Fig. 5). The taxonomic composition and abundances of MAGs at the phylum level were similar to the results obtained from 16 S rRNA gene amplicon sequencing (Supplementary Figs. 4c, 6), suggesting that MAGs were representative of total bacterial diversity. More specifically, the relative abundances of *Flavobacteriaceae* (including *Flavobacterium*, *Sediminicola*, *Gelidibacter*, *Aequorivita,* and *Galbibacter*) and *Paenibacillaceae* (including *Paenibacillus*) in both WT and *nrh* plants were higher in acid soil than in alkaline soil under MSD based on metagenomics (Fig. 2c and Supplementary Data 2). The abundances of these MAGs were also significantly enriched in the barley rhizosheath compared to bulk soil (Fig. 2c; Supplementary Data 2). Next, the functional genes of MAGs within *Flavobacteriaceae* and *Paenibacillaceae* were annotated based on the Kyoto Encyclopedia of Genes and Genomes (KEGG) Orthology database. Interestingly, pathways linked to indole-3-acetic acid (IAA, a commonly occurring auxin) biosynthesis in rhizosheath in acid soil were significantly enriched relative to those in alkaline soil (Supplementary Data 3). As expected, several IAA biosynthesis related proteins were found in the *Flavobacteriaceae* and *Paenibacillaceae* genomes obtained using metagenomics (Supplementary Data 4). To explore the activities of *Flavobacteriaceae* and *Paenibacillaceae* in the rhizosheath, we mapped metatranscriptomic reads against assembled MAGs. The transcriptional activities of *Flavobacteriaceae* (including *Aequorivita*, *Flavobacterium*, *Galbibacter*, *Chryseobacterium*, *Gelidibacter* and *Sediminicola*) and *Paenibacillaceae* (including *Paenibacillus*) were significantly higher in acid soil than alkaline soil under MSD (Fig. 2d). Additionally, these bacteria were more active in the barley rhizosheath compared to bulk soil (Fig. 2d). These results suggested that *Flavobacteriaceae* and *Paenibacillaceae* were important for rhizosheath formation, perhaps due to IAA.

### Roles of *Flavobacteriaceae* and *Paenibacillaceae* in barley rhizosheath formation

**Fig. 3:**
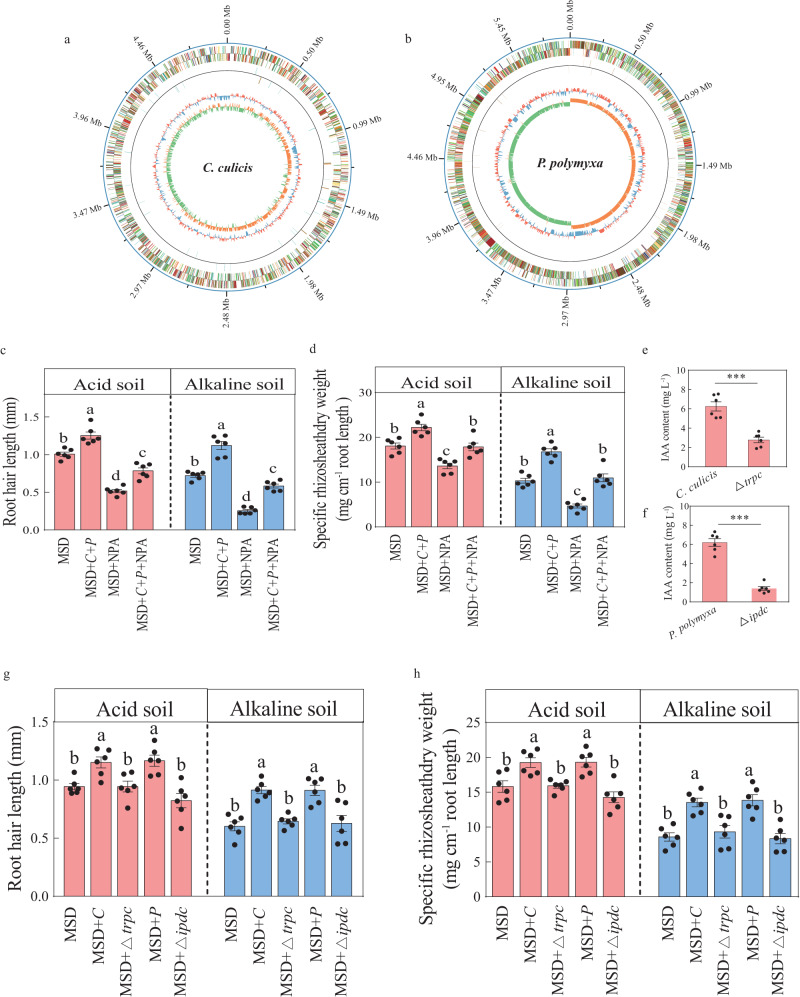
Barley rhizosheath formation is increased by *C. culicis* and *P. polymyxa* inoculation through auxin (IAA) production under moderate soil drying (with Supplementary Fig. 7, Supplementary Data 5–7 and Supplementary Tables 2, 4). **a**, **b** Overview of *C. culicis* (a) and *P. polymyxa* (**b**) genomes. The circles represent (from outside to inside): circle 1, genome size; circle 2 and 3, genes existing in the genome with different Cluster of Orthologous Groups of proteins (COG) function; circle 4, ncRNA; circle 5, GC-content; circle 6, GC-skew. **c**, **d** Root hair length (**c**) and rhizosheath formation (**d**) of barley not inoculated (MSD), co-inoculated with *C. culicis* and *P. polymyxa* under MSD (MSD + *C* + *P*), with NPA (an auxin transport inhibitor) under MSD (MSD + NPA) and co-inoculated with *C. culicis* and *P. polymyxa* under MSD with NPA (MSD + *C* + *P* + NPA). Data are means ± SE (*n* = 6 independent replicates). Bars with different letters indicate significant differences among treatments at *p* < 0.05 (one-way ANOVA, Tukey’s HSD, two-sided). **e**, **f** IAA content of the *C. culicis* and △*trpc* (**e**) or *P. polymyxa* and △*ipdc* (**f**). Data are means ± SE (*n* = 6 indepe*n*dent replicates). Asterisks indicate significant differences (***p < 0.001) by two-sided Student’s *t* test. **g**, **h** Root hair length (**g**) and rhizosheath formation (**h**) of barley not inoculated (MSD), inoculated with *C. culicis* under MSD (MSD + *C*), inoculated with △*trpc* under MSD (MSD + △*trpc*), inoculated with *P. polymyxa* under MSD (MSD + *P*) and inoculated with △*ipdc* under MSD (MSD + △*ipdc*). Data are means ± SE (*n* = 6 independent replicates). Bars with different letters indicate significant differences among treatments at *p* < 0.05 (one-way ANOVA, Tukey’s HSD, two-sided). The exact *p* values are provided in the Source Data file.

To further assess the roles of *Flavobacteriaceae* and *Paenibacillaceae* in rhizosheath formation, 224 strains were isolated, 113 and 111 of which were obtained from rhizosheath in acid and alkaline soils, respectively (Supplementary Table 2). Two strains of *Flavobacteriaceae* (*Chryseobacterium culicis*) and *Paenibacillaceae* (*Paenibacillus polymyxa*) isolated exclusively from the rhizosheath in acid soil were selected for next analysis (Supplementary Table 2). Both strains had the capacity to produce IAA (Fig. 3c, d). To conform that *C. culicis* and *P. polymyxa* can promote rhizosheath formation through IAA production, we performed whole genome sequencing of the two strains (Fig. 3a, b). The genome size of *C. culicis* was approximately 4,945,394 bp and the GC content was 36.2% (Fig. 3a). The genome size of *P. polymyxa* was approximately 5,895,306 bp and the GC content was 45.6% (Fig. 3b). Several IAA biosynthesis related genes were found in the *C. culicis* and *P. polymyxa* genomes (Supplementary Data 5, 6). Then, we applied *C. culicis* and *P. polymyxa* to barley in acid and alkaline soils to assess the effect of them on rhizosheath formation under MSD (Fig. 3c, d). Significant increases in root hair length and rhizosheath formation were observed after co-inoculation with *C. culicis* and *P. polymyxa* under MSD compared to MSD without bacterial inoculation in acid or alkaline soil (Fig. 3c, d). Barley rhizosheath formation treated with *C. culicis*, *P. polymyxa* and 1-naphthylphthalamic acid (NPA, a polar auxin transport inhibitor) was significantly reduced in acid or alkaline soil compared to barley co-inoculated with *C. culicis* and *P. polymyxa* (Fig. 3c).

Besides some tryptophan synthase genes, we found that the indole-3-pyruvic acid pathway (IPyA) is main IAA metabolic pathway in both *C. culicis* and *P. polymyxa* (Supplementary Data 5, 6). To further investigate whether auxin-producing microbiota associates with barley rhizosheath, we successfully performed mutants by deleting the indole-3-glycerol phosphate synthase gene (*trpC*) of tryptophan synthase in *C. culicis* and the indole-3-pyruvate decarboxylase gene (*ipdC*) of IPyA in *P. polymyxa* (Supplementary Fig. 7a, b and Supplementary Data 7a, b). The IAA-producing capability of △*trpc* and △*ipdc* was significantly reduced compared to WT *C. culicis* and *P. polymyxa*, respectively (Fig. 3e, f). To explore the effect of the WT (*C. culicis* and *P. polymyxa*) and IAA mutants (△*trpc* and △*ipdc*) on barley rhizosheath formation, the strains were inoculated in acid or alkaline soil under MSD (Fig. 3g, h). In acid soil, significant increases in root hair length and rhizosheath formation of barley were observed after inoculation with WT *C. culicis* strain under MSD compared to MSD without bacterial inoculation (Fig. 3g, h). Difference of root hair length and rhizosheath formation in barley inoculated with the △*trpc* was not significant compared to MSD without bacterial inoculation (Fig. 3g, h). In alkaline soil, the increases in root hair length and rhizosheath formation of barley plants were also significant after inoculation with WT *C. culicis* strain under MSD compared to MSD without bacterial inoculation (Fig. 3g, h). There was no significant difference in root hair length and rhizosheath formation of barley between △*trpc* inoculation and non-inoculation treatments in alkaline soil (Fig. 3g, h). In addition, rhizosheth formation of barley inoculated with *P. polymyxa* under MSD was significantly greater than MSD without bacterial inoculation in acid soil, while that of barley inoculated with △*ipdc* was not significant (Fig. 3h). In alkaline soil, root hair length and rhizosheath formation of barley inoculated with *P. polymyxa* under MSD were significantly increased compared to MSD without bacterial inoculation (Fig. 3g, h). However, no significant difference in root hair length and rhizosheath formation was recorded between MSD and MSD with △*ipdc* inoculation in alkaline soil (Fig. 3g, h). Rhizosheath formation in barley with △*trpc* or △*ipdc* inoculation was also significantly reduced compared to WT *C. culicis* or *P. polymyxa*, respectively (Fig. 3g, h). Together, these results showed that *C. culicis* and *P. polymyxa* can promote rhizosheath formation, and this is associated with IAA production.

### Co-inoculation with *C. culicis* and *P. polymyxa* promoted barley grain yield

**Fig. 4:**
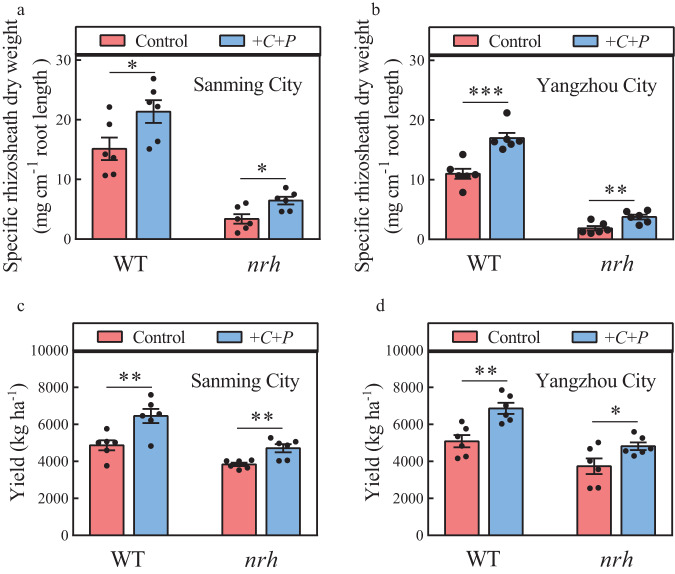
Barley rhizosheath formation and grain yield are increased by *C. culicis* and *P. polymyxa* inoculation in the field (with Supplementary Fig. 8 and Supplementary Table 3). **a**, **b** Rhizosheath formation of barley plants not inoculated (control) and co-inoculated with *C. culicis* and *P. polymyxa* (+*C* + *P*) in Sanming City (**a**, 118°29′E, 26°17′N) and Yangzhou City (**b**, 119°25′E, 32°23′N). **c**, **d** Grain yield of barley plants not inoculated (control) and co-inoculated with *C. culicis* and *P. polymyxa* (+*C* + *P*) in Sanming City (**c**) and Yangzhou City (**d**). Data are means ± SE (*n* = 6 plots). Asterisks indicate significant differences between treatments (*p < 0.05, **p < 0.01, ***p < 0.001) by two-sided Student’s *t* test. The exact *p* values are provided in the Source Data file.

To verify the effects of *C. culicis* and *P. polymyxa* on barley rhizosheath formation and grain yield, we conducted field trials in Yangzhou City (119°25′E, 32°23′N) and Sanming City (118°29′E, 26°17′N). Compared to the non-inoculated controls, the rhizosheath formation of WT and *nrh* plants were significantly increased with *C. culicis* and *P. polymyxa* co-inoculation (Fig. 4a–d). At the two locations, grain yield of WT and *nrh* plants co-inoculated with *C. culicis* and *P. polymyxa* was 32.6–34.9% and 22.6–29.0%, respectively, higher than the non-inoculated controls (Fig. 4a–d). The higher yield was correlated with an increase in spike number (36.7–42.9% in the WT and 12.7–21.5% in the *nrh*; Supplementary Fig. 8a, b). Plant height, spike length, grain number per spike, filled grain rate, thousand kernel weight, grain length, grain width, and harvest index were similar between the non-inoculated control and co-inoculated WT and *nrh* plants (Supplementary Table 3). Moreover, a significant positive linear relationship was found between rhizosheath formation and barley grain yield at the two locations (Supplementary Fig. 8c, d). These results showed that *C. culicis* and *P. polymyxa* promoted barley grain yield in field trials.

## Discussion

### Microbiota is important for barley rhizosheath formation in acid or alkaline soil

The rhizosheath can encase the total root system^4,6,11,24^. And, the rhizosheath is an important adaptive-trait for crops under drought stress and contributes to agricultural sustainability^6,34^. In our study, barley rhizosheath formation was significantly increased under MSD compared to that under WW (Fig. 1e, f and Supplementary Fig. 1b), consistent with previous findings in other plant species^10,11,21,26^. Root hair is associated with maintenance of wheat rhizosheath formation in acid soil^19^. Barley rhizosheath formation in acid soil was significantly higher than that in alkaline soil (Fig. 1e, f), possibly related to differences of root hairs^19^. The difference in rhizosheath formation between acid soil and alkaline soil was not significant using sterilized soils (Fig. 1e, f), implicating that soil microorganisms are the important factor for rhizosheath formation^4^. Moreover, the increase in rhizosheath formation by addition of live microbiota from the rhizosheath of acid soil to alkaline soil was detected, which confirms the important effects of microbiota in barley rhizosheath formation (Fig. 1g, h). These results suggest that microbiota plays a key roles in barley rhizosheath formation in acid or alkaline soil.

### *Flavobacteriaceae* and *Paenibacillaceae* promote barley rhizosheath formation, and this is associated with IAA production

The rhizosheath is a “hot spot” for plant microbiota, which had plant growth-promoting functions and was involved in plant growth promotion under stress conditions^35^. It generates a favorable microenvironment for bacterial colonization^25,36^. In our study, plant growth-promoting rhizobacterias (PGPRs) like *Flavobacteriaceae* and *Paenibacillaceae* were enriched in the rhizosheath in acid soil compared with alkaline soil under MSD or WW (Fig. 2a–d and Supplementary Data 1d), suggesting the importance of PGPRs for barley rhizosheath formation^11^. Furthermore, the *Flavobacteriaceae* (including *Aequorivita*, *Flavobacterium*, *Galbibacter*, *Chryseobacterium*, *Gelidibacter* and *Sediminicola*) and *Paenibacillaceae* (including *Paenibacillus*) reconstructed from metagenomic showed potential function of IAA biosynthesis (Fig. 2c and Supplementary Data 3), which suggests that the *Flavobacteriaceae* and *Paenibacillaceae* may increase barley rhizosheath formation by IAA pathway. The transcriptional activities of *Flavobacteriaceae* (including *Aequorivita*, *Flavobacterium*, *Galbibacter*, *Chryseobacterium*, *Gelidibacter,* and *Sediminicola*) and *Paenibacillaceae* (including *Paenibacillus*) in acid soil were significantly enhanced relative to alkaline soil (Fig. 2d), which also provides an important evidence for barley rhizosheath formation by *Flavobacteriaceae* and *Paenibacillaceae*. Moreover, the rhizosheath formation in barley plants was significantly decreased when the auxin transport inhibitor NPA was added (Fig. 3d), which also suggests that *Flavobacteriaceae* and *Paenibacillaceae* could promote barley rhizosheath formation, perhaps due to IAA production.

*Chryseobacterium gleum* enhancing wheat growth via IAA production has been reported previously^37^. In our study, *C. culicis* and *P. polymyxa* exclusively isolated from the rhizosheath of acid soil (Supplementary Table 2), and they had plant growth-promoting function by generating IAA (Fig. 3e, f), which is consistent with a report that *P. polymyxa* promotes plant growth by triggering plant hormone producation^38^. Tryptophan is the main precursor in microbial IAA biosynthesis^39^. Therefore, we generated a mutant of indole-3-glycerol phosphate synthase gene (*trpC*), an important gene in tryptophan biosynthesis^40,41^, in *C. culicis* (Fig. 3e; Supplementary Fig. 7a and Supplementary Data 7a). It is reported that indole-3-pyruvate decarboxylase (IPDC), a key enzyme in the IPyA pathway, is necessary for IAA production in *P. polymyxa*^42^. In our study, the *ipdC*, which encodes IPDC, was also deleted in *P. polymyxa*. (Fig. 3f; Supplementary Fig. 7b and Supplementary Data 7b). The IAA content of △*trpc* and △*ipdc* was significantly decreased relative to *C. culicis* and *P. polymyxa* (Fig. 3e, f), but it does not eliminate IAA production. The results suggest that, besides IPyA pathway, there are some other IAA synthesis pathways in *C. culicis* and *P. polymyxa*. Furthermore, inoculation with △*trpc* or △*ipdc* significantly decreased barley rhizosheath formation under MSD compared to *C. culicis* or *P. polymyxa* due to short root hair (Fig. 3g, h), which demonstrates that the promotion of rhizosheath formation by *C. culicis* and *P. polymyxa* is dependent on bacterial-derived IAA production. This is probably because there may be a dose response relationship between bacterial auxin production and the rhizosheath formation. These results suggest that the *C. culicis* and *P. polymyxa* are enriched in rhizosheath, produce IAA for root hair growth, and thereby enhance rhizosheath formation for water use (Supplementary Fig. 9).

### *C. culicis-* and *P. polymyxa-*induced rhizosheath formation enhances barley grain yield in the field trails

Rhizosheath contributes substantially to water use and crop performance. Barley rhizosheath formation was significantly increased by *C. culicis* and *P. polymyxa* co-inoculation in the field conditions (Fig. 4a, b). The probable reason for the increase was the enhanced barley rhizosheath formation (Fig. 3c, d). It is reported that barley genotypes with large rhizosheaths show increased tiller numbers and grain yields under drought stress compared to the barley with small rhizosheaths^24^. Rhizosheath formation can contribute to the maize grain yield^43^. Moreover, rhizosheath formation and barley biomass are significantly related under phosphorus and drought stresses^17^. In our study, barley with a large rhizosheath (co-inoculation with *C. culicis* and *P. polymyxa*) showed a greater grain yield than the barley with a small rhizosheath (no-inoculation controls) (Fig. 4a–d), which suggests that the barley rhizosheath can also contribute to barley grain yield. A significant positive linear relationship between barley rhizosheath formation and barley grain yield in the two locations was also analyzed (Supplementary Fig. 8c, d), likely because the rhizosheath can improve water and nutrient uptake^4,15^. Importantly, co-inoculation with *C. culicis* and *P. polymyxa* further improved barley yield by increasing spike number at both locations (Fig. 4d, e; Supplementary Fig. 8a, b and Supplementary Table 3). These results suggest that *C. culicis-* and *P. polymyxa-*induced rhizosheath formation can enhance barley grain yield in the field conditions. In addition, *Flavobacteria* strain showed positive effects on growth of tomato and lupine^44,45^. *Paenibacillus polymyxa* strain can promote plant rice growth by enhancing the synthesis of IAA^46^. The results suggest that our study is more generalizable for rhizosheath formation.

In conclusion, our results revealed that barley rhizosheath formation is increased by the IAA production in *C. culicis* and *P. polymyxa*, which also promotes barley grain yield (Supplementary Fig. 9). These results provide insights into barley rhizosheath formation and suggest new approaches to promote barley grain yield in acid or alkaline soil, which contributes to plant adaptation under the climate change.

## Methods

### Plant materials

Barley (*Hordeum vulgare*. L) cv Optic (WT) and its root hair lacking mutant *no root hair* (*nrh*)^22,24^ were used in this study. Seeds were surface-sterilized using 1.5% (v/v) NaClO for 20 min, rinsed five times with double-distilled water, and placed on moistened filter paper at 4 °C in the dark for 3 days. Next, the seeds were grown for 3 d on moistened filter paper under a 14 h light (26 °C)/10 h dark (22 °C) cylce, 60% (w/w) relative humidity, and a photosynthetic photon flux density of 300 mmol photons m^−2^ s^−1^. Seedlings of uniform size were transplanted into pots (12 cm diameter, 14 cm height), which contained 1.8 kg of dry soil from a paddy field in Huayang, China (acid soil; 115°09′E, 28°32′N) and Ronghuashan, China (alkaline soil; 122°86′E, 39°93′N) (Fig. S1A). The air-dried soil with mineral nutrients added was sieved through a 4 mm mesh to remove any coarse material and vegetative matter. The chemical properties of the two soils are listed in Supplementary Table 1. For the WW treatment, half of the pots were watered with 200 mL of water every 2 d. For the MSD treatment, seedlings were irrigated with 400 mL water every 6 d^35^. The pots were distributed in a random arrangement in the greenhouse. For the sterilized soil, it was sterilized three times by autoclaving and heat-incubation until completely dehydrated^47^. For the treatments with the auxin efflux inhibitor 1-naphthylphthalamic acid (NPA), 10 μM of NPA was used to evaluate barley phenotype and rhizosheath formation under MSD^21^. The experiments were conducted in the greenhouse under a 14 h light (26 °C)/10 h dark (22 °C) cycle, 60% (w/w) relative humidity, and a photosynthetic photon flux density of 300 mmol photons m^−2^ s^−1^.

### Determination of root phenotypic traits

Under MSD, barley roots were carefully shaken after the pots had been disassembled. Soil that tightly adhered to roots upon excavation, was defined as rhizosheath soil^4,11,17,21^. In brief, roots together with closely attached soil were washed with double-distilled water in plastic dishes. The washed soil and rinsed water were dried at 105 °C for 3 d to determine the soil dry weight. Total root length was measured using an Expression 1640XL flat-bed scanner (Epson UK, London, UK) and WinRHIZO software (Regent Instruments, Quebec City, QC, Canada). The specific rhizosheath dry weight was calculated as the dry weight of attached soil (mg) per unit total root length (cm)^23^. Root hair length was measured according to George et al. ^17^. Photographs were obtained using a SMZ18 stereomicroscope (×5 magnification) with a DS-U3 camera (Nikon). Ten fully elongated root hairs were measured an average root hair length per root using Image J software (National Institutes of Health, Bethesda, MD, USA; v 1.8.0)^48^. The ten measurements across the root samples were averaged and used as single value for each sample^27,48^. Three replicates for the barley plants were selected for the experimental measurement, and the experiments were repeated two times.

### Rhizosheath transplantation between two soil types

To investigate the effect of the rhizosheath of acid soil (much rhizosheath formation) on alkaline soil (little rhizosheath formation), we examined root hair morphology and rhizosheath formation by a transplantation strategy as described previously^49^. In brief, barley plants were grown in acid and alkaline soils under MSD. Next, the rhizosheath was collected (as in the “Determination of root phenotypic traits” section) and sterile water was added to create acid and alkaline rhizosheath soil slurries. The slurries were used to inoculate barley for determination of rhizosheath formation in alkaline or acid soils (Supplementary Fig. 2). Slurry sterilized by autoclaving (121 °C, 1 h) three times as the control. After 18 d, barley root hair length and rhizosheath formation were determined. Three replicates for the barley plants were selected for the experimental measurement, and the experiments were repeated two times.

### Soil sample preparation and DNA extraction

Collection of samples for DNA extractions was performed according to Prendergast et al.^50^ and Zhang et al.^23^. Briefly, root and rhizosheath samples were harvested under both WW and MSD conditions. Root samples were cleaned by washing with PBS-S buffer in 50-mL Falcon tubes^51^. Next, rhizosheath soil was collected from the wash buffer by centrifugation at 1500 × *g* for 20 min at 4 °C. Bulk soil was taken from the pots without plant treatments. After collection, soil samples were immediately frozen in liquid nitrogen and stored at −80 °C. Washed root samples were surface-sterilized with 1.5% (v/v) NaClO for 15 min and washed three times with sterilized double-distilled water. Thereafter, the final washed water was used to verify the sterilization efficacy by incubating the water on Luria-Bertani (LB) plates^52^. Finally, sterilized root samples were stored at −80 °C for next analysis. Total root and soil genomic DNA was extracted from 0.5 g samples using the Mag-Bind Soil DNA Kit (Omega Bio-Tek) following manufacturer’s instructions. DNA quality and quantity were determined by gel electrophoresis and NanoDrop ONE spectrophotometry (Thermo Scientific, Waltham, MA, USA). Three repeats of each treatment were taken for next high-throughput sequencing.

### 16 S rRNA gene amplicon sequencing for bacterial community analysis

All collected DNA samples were subjected to 16 S rRNA gene amplicon sequencing targeting the V5-V7 hypervariable region using the primers 799 F (5’-AACMGGATTAGATACCCKG-3’)^53^ and 1193 R (5’-ACGTCATCCCCACCTTCC-3’)^54^ using a NovaSeq6000 platform (Illumina). DADA2 was used to quality-filter (i.e., filtered, dereplicated, denoised, merged, and assessed for chimaeras) the raw 16 S rRNA gene amplicon sequencing reads via QIIME2^55^. Mitochondria- and chloroplast-assigned ASVs were deleted. Next, the DADA2 generated feature table was filtered to delete ASVs at a frequency less than two^56^. ASVs were classified using the QIIME2 naive Bayes classifier trained on 99% operational taxonomic units against SILVA (v 138)^57^. Microbial diversity was estimated using alpha-diversity (Chao) and community composition using the beta-diversity based on the q2-diversity pipeline within QIIME2. LEfSe was performed to detect taxa that different significantly (*p* < 0.05) among treatments. For principal coordinates analysis (PCoA), PERMANOVA (Adonis function, 999 permutations) was used to evaluate the bacterial community composition based on the Bray–Curtis distance.

### Metagenomic sequencing and data analysis

To explore the functional capacity of the rhizosheath microbial community, soil samples collected from the rhizosheath of WT, *nrh,* and bulk soil under MSD were subjected to shotgun metagenomic sequencing on an Illumina NovaSeq6000 sequencer (Illumina, PE150) at Majorbio Bio-Pharm Technology Co. Ltd (Shanghai, China). DNA quality was assessed with a 1% agarose gel and DNA concentration was measured with Qubit dsDNA high-sensitivity assays (Thermo Fisher, Waltham, MA, USA). Libraries were prepared using the NEB Next Ultra DNA Library Prep Kit for Illumina (New England Biolabs, MA, USA) according to the manufacturer’s instructions. This approach yielded 1,850,574,708 reads (average 51,404,853 reads/sample) for construction of the metagenome. Trimmomatic (v 0.39, score > 30 and length >36 bases) was used for raw data processing^58^ and Bowtie2 (v 2.5.0)^59^ software was used to remove possible eukaryotic genome sequences with the “--very-sensitive” parameter. Then, the contigs were assembled separately using SPAdes v 3.13.1 with the parameter “-k 33, 55, 77, 99, 111, 127 --meta”^60^. Assembled contigs longer than 2.0 kb were binned using MetaWRAP^61^ based on MetaBAT2^62^, MaxBin2^63^ and Concoct^64^ with the default parameters. Bins were further curated to obtain high-quality genomes using the Bin_refinement module in MetaWRAP^61^. The quality of MAGs was assessed using CheckM (v 1.0.13)^65^ and MAGs with greater than 50% completeness and less than 10% contamination were retained for further analyses. MAGs from different samples were dereplicated using dRep v 2.3.2^66^ and assigned to taxonomic classifications based on the Genome Taxonomy Database (GTDB; release 03-RS86) using the GTDB-Tk toolkit (v 0.3.2) with the classify workflow^67^. The relative abundances of MAGs were quantified based on the coverage of mapped reads using the CoverM pipeline^68^ (v 0.61, https://github.com/wwood/CoverM) in ‘genome’ mode. Briefly, reads were first mapped to MAGs using “make” command to create BAM files (--percentage_id 0.95 --percentage_aln 0.75). Filtered BAM files were then used to generate coverage profiles across samples (--trim-min 0.10 --trim-max 0.90 --min-read-percent-identity 0.95 --min-read-aligned-percent 0.75 -m mean). RPKM (reads per kilobase of exon per million reads mapped) is used for relative abundance with metagenomic datasets^69^. Prodigal (v 2.6.3) was used to predict open reading frames (ORFs) longer than 100 bp using the default parameters^70^. The CD-HIT (v 4.8.1) tool was employed to remove redundancy and obtain a catalog of unigenes (i.e., nucleotide sequences encoded by unique and continuous genes) with the default parameters of -c 0.95 -aS 0.8^71^. The longest sequences in each catalog were chosen to be the representative sequences. Then, the gene catalogs were mapped to clean data using BBMap (v 38.90) with the default parameters to determine the abundance of genes in each sample. Functional genes were annotated through matching with the functional gene databases KEGG (Release 101.0)^72^ and eggNOG 5.0^73^ using DIAMOND^74^ with an e-value criterion of ≤0.001. The merge heatmap of relative abundance was generated by TBtools (v 1.120)^75^. Three repeats of each treatment were used for metagenomic sequencing.

### Metatranscriptomic sequencing and data analysis

The rhizosheath of WT, *nrh,* and bulk soil under MSD were selected for RNA shotgun sequencing. Total genomic RNA was extracted from 0.5 g samples using the RNeasy PowerSoil total RNA kit (Qiagen) according to the manufacturers’ instructions. RNA concentrations were measured using the Qubit RNA HS assay kit and RNA integrity was determined using an Agilent 2100 Bioanalyzer (Agilent Technologies) before and after rRNA removal with Ribo-minus Transcriptome Isolation Kit (Thermo Fisher). The resulting enriched mRNA was prepared for sequencing using the TruSeq stranded mRNA library prep kit (Illumina, San Diego, CA, USA), according to the manufacturer’s instructions. The extracted RNA and cDNA from each sample were used for library construction at Majorbio Bio-Pharm Technology Co. Ltd using the NovaSeq6000 platform. Metatranscriptomic reads were quality filtered using Trimmomatic (v 0.39)^68^. Non-coding rRNA sequences were removed from the metatranscriptomic reads using SortMeRNA (v 4.3.4)^76^. The mRNA reads were then mapped to the barley reference genome (MorexV3) using Bowtie2 (v 2.5.0)^59^ to filter potential host RNA contaminations. Then, the mRNA reads were mapped to contigs to identify active bacterial taxa using Minimap2^77^ in the CoverM pipeline (https://github.com/wwood/CoverM). Briefly, metatranscriptomic datasets were used as input reads, with the same mapping parameters as metagenomic read mapping, except for the “tpm” calculation method (transcripts per kilobase per million mapped reads, TPM). Bacteria were deemed active when TPM values were greater than 0. To assess the expression of annotated genes in assembled MAGs, mRNA reads were mapped to a concatenated Fasta file containing all genes of MAG bin using HISAT2 with the default parameters^78^. Quantification of mapped reads per identified gene was performed with the function featureCounts of the R Subread package^79^. The transcript abundance of each gene was converted to transcript number per million reads at each sampling depth. The merge heatmap of transcript activity was generated by TBtools (v 1.120)^75^.

### Bacterial culture and isolation

To isolate the putative strains from rhizosheath soil, fresh soil was suspended in diluent (NaCl 4.25 g L^−1^, KH_2_PO_4_ 0.15 g L^−1^, Na_2_HPO_4_ 0.3 g L^−1^, MgSO_4_ 0.1 g L^−1^, gelatin 0.05 g L^−1^), and then plated on 0.5× TSA (0.5× TSA; 7.5 g L^−1^ tryptone, 2.5 g L^−1^ soytone, 2.5 g L^−1^ sodium chloride, and 15 g L^−1^ agar, pH 7.0)^80^. After incubation for 48 h at 30 °C, colonies were randomly isolated from the plates. A total of 224 strains were isolated, 113 and 111 from the acid and alkaline rhizosheath, respectively (Supplementary Table 2). Bacterial colonies were identified at the species level by sequencing 16 S rRNA gene using the primers 27 F and 1492 R (Supplementary Table 4). Next, we aligned the sequence reads using BLASTn, and identified the closest match.

### Genome sequencing of *C. culicis* and *P. polymyxa*

Genomic DNA of *C. culicis* and *P. polymyxa* was extracted using the SDS method. Then, the DNA was measured using agarose gel electrophoresis and assessed using a Qubit®2.0 Fluorometer (Thermo Scientific, Waltham, MA, USA). Purified genomic DNA was used to construct a sequencing library with the NEB Next® Ultra^TM^ DNA Library Prep Kit (NEB, Beverly, MA, USA). The genomes of *C. culicis* and *P. polymyxa* were sequenced on the Nanopore PromethION platform and Illumina NovaSeq PE150 (Beijing Novogene Bioinformatics Technology Co., Ltd, Beijing, China). After trimming low-quality reads using fastq, clean reads were assembled using SPAdes 3.13.1^59,81^. Bioinformatics analysis focused on KEGG Orthology^82^. Genome overviews were created by Circos to reveal the annotations^83^.

### IAA concentration determination and IAA biosynthesis mutant construction

The level of IAA concentration in *C. culicis* and *P. polymyxa* were determined as described^84^. Briefly, Landy medium^85^ was used to culture strains for 24 h and 200 rpm min^−1^ at 30 °C. Supernatants were obtained by centrifugation at 10,000 × *g* for 15 min with addition of 100 µL 10 mM orthophosphoric acid and 4 mL reagent (1 mL of 0.5 M FeCl_3_ in 50 mL of 35% HClO_4_) and incubated in darkness at room temperature for 25 min. The absorbance of pink color developed was obtained at 530 nm. A calibration curve of pure IAA was used to determine the IAA concentration in culture. Three replicates for the strains were selected for the experimental measurement, and the experiments were repeated two times.

Based on the genome sequences of *C. culicis* and *P. polymyxa*, mutants of the IAA biosynthesis genes *trpC* and *ipdC* were generated using the primers listed in Supplementary Table 4. The suicide vector pRE112 was used to generate IAA production mutants of *C. culicis* and *P. polymyxa* according to Yao et al. ^86^. Upstream and downstream of *trpC* and *ipdC* were amplified from the genomic DNA of *C. culicis* and *P. polymyxa*. Next, the segments were ligated by overlapping PCR, and the resulting target was inserted into the vector pRE112 using the *XbaI* restriction site. Recombinant plasmids were transformed successively into *E. coli* MC1061 and *E. coli* S17-1 cells. The plasmids were transferred via conjugation to *C. culicis* and *P. polymyxa* to select mutant colonies, which were confirmed by PCR and Sanger sequencing.

### Study of *C. culicis* and *P. polymyxa* on barley rhizosheath formation

To assess the effect of *C. culicis*, *P. polymyxa* and IAA biosynthesis mutant △*trpc*, △*ipdc* on barley growth and rhizosheath formation, half of the seedlings were inoculated with suspensions of the strains to 10^8^ cells g^−1^ soil. Other seedlings were watered with sterilized double-distilled water^87^. After 7 d of inoculation, the seedlings were subjected to MSD for 18 days, as described in the “Plant materials” section. Total root length, root hair length, and rhizosheath weight were determined as described in the “Determination of root phenotypic traits” section. Three replicates for the barley plants were selected for the experimental measurement, and the experiments were repeated two times.

### Study of *C. culicis* and *P. polymyxa* on barley growth in the field conditions

Barley plants were grown from the winter of 2021 to the spring of 2022 at two controlled experimental stations in the field experimental station of Fujian Agriculture and Forestry University (Fujian Province, China; 118°29′E, 26°17′N) and the field experimental station of Yangzhou University (Jiangsu Province, China; 119°25′E, 32°23′N). The soil (pH 6.3) in the field experimental station of Fujian Agriculture and Forestry University contained 25.02 g kg^−1^ organic C, 84.7 mg kg^−1^ available N, 25.4 mg kg^−1^ available P, and 68.7 mg kg^−1^ available K. The soil (pH 7.2) in the field experimental station of Yangzhou University contained 21.6 g kg^−1^ organic C, 105.5 mg kg^−1^ available N, 35.6 mg kg^−1^ available P, and 78.1 mg kg^−1^ available K. Plots were fertilized at rates of 100 kg ha^−1^ N, 90 kg ha^−1^ P_2_O_5_ and 150 kg ha^−1^ K_2_O to avoid deficiencies of those elements. Barley plants were planted in a spilt plot design with plots arranged in six replicates of randomized complete blocks. The plot size was 1 m wide × 2 m long, and row spacing was 0.25 m. To eliminate surface-associated microbes, barley seeds were surface-sterilized in 75% ethanol for 1 min and 1.2% sodium hypochlorite for 10 min and washed five times in sterile water. Barley seeds were planted at ~300 seeds m^−2^. For inoculation, *C. culicis* and *P. polymyxa* were cultured in growth medium at 30 °C with shaking at 200 rpm for 24 h. The mixed suspension (in 50 μM PBS, pH 7.0, OD600 = 1.0) was directly inoculated to 4-week-old barley plants and soil. As the control, the same amount of PBS buffer was added. Fungicides and insecticides were sprayed to control pests and diseases, and weeds were periodically removed by hand. After one month of inoculation, total root length, root hair length, and rhizosheath weight were determined. At maturity, 1 m^2^ of plants were selected at the center of each plot and harvested for determination of shoot biomass, grain yield, and yield components.

### Statistical analysis

Graphical representations were generated with Prism 7.0 (GraphPad Software, Inc., La Jolla, CA, USA). Means and standard error (SE) of data were calculated. Significant differences were determined using SPSS v. 20.0 (IBM Corporation, NY, USA). *p* < 0.05 was considered significant for both statistical methods. Tukey’s HSD and the two-sided Student’s *t* test were used to analyze the differences between treatments. The Kruskal–Wallis (KW) sum-rank test was used to identify features displaying significantly different abundances between assigned families in LEfSe analysis.

### Reporting summary

Further information on research design is available in the Nature Portfolio Reporting Summary linked to this article.

### Supplementary information

Supplementary Information

Peer Review File

Description of Additional Supplementary Files

Supplementary Dataset 1–6

Supplementary Dataset 7

Reporting Summary

### Source data

Source Data

## Data Availability

16 S rRNA gene amplicon sequencing data for the study have been uploaded to the NCBI SRA (https://www.ncbi.nlm.nih.gov) under accession number: PRJNA867556. The metagenome and metatranscriptome sequencing data are deposited in Genome Sequence Archive (GSA, https://ngdc.cncb.ac.cn/) in the BIG Data Center, Chinese Academy of Science under BioProject accessions PRJCA016632 and PRJCA016646. The genome of *P. polymyxa* is deposited in NCBI under BioProject accessions PRJNA908138. The genome of *C. culicis* is deposited in GSA under BioProject accessions PRJCA016210. The source data are provided as a Source Data file. Source data are provided with this paper.

## Code availability

All codes used in the study are available at GitHub (https://github.com/xufychallenge/xufy-NC.git).
