# Supplementary material for: Auxin-producing bacteria promote barley rhizosheath formation
Source: Nat Commun. 2023 Sep 19;14:5800. doi: 10.1038/s41467-023-40916-4 (PMC10509245; doi:10.1038/s41467-023-40916-4)
Supplement: Supplementary file 1 — Supplementary Information [file 41467_2023_40916_MOESM1_ESM.pdf]

# Auxin-producing bacteria promote barley rhizosheath formation

Feiyun Xu[1,#], Hanpeng Liao[2,#], Jinyong Yang[1,#], Yingjiao Zhang[1,3,#], Peng Yu[4,5,#], Yiying Cao[1], Ju Fang[1], Shu Chen[1], Liang Li[1], Leyun Sun[1], Chongxuan Du[1], Ke Wang[1], Xiaolin Dang[1], Zhiwei Feng[1], Yifan Cao[6], Ying Li[6], Jianhua Zhang[7], Weifeng Xu[1,*]

**Supplementary Table 1 Soil chemical factors of sampling sites.**

| Soil component | Acid soil | Alkaline soil |
|---|---|---|
| pH | 5.4 | 7.5 |
| OM | 26.4 | 23.5 |
| TN | 1.38 | 1.85 |
| TP | 0.65 | 0.57 |
| TK | 27.7 | 20.87 |
| AP | 42.6 | 55.26 |
| AK | 92 | 78 |

pH, potential of hydrogen, OM, organic matter (mg/kg); TN, total nitrogen (g/kg); TP, total phosphorus (g/kg); TK, total potassium (g/kg); AP, Olsen-P (mg/kg); AK, exchange K (mg/kg).

**Supplementary Table 2 Taxonomy of isolates collected from barley rhizosheath of acid soil and alkaline soil under MSD.**

| Samples | Genus | Species | Number of isolates |
|---|---|---|---|
| Acid soil | *Agrobacterium* | *Agrobacterium fabrum* | 4 |
| | *Agrobacterium* | *Agrobacterium tumefaciens* | 2 |
| | *Bacillus* | *Bacillus cucumis* | 1 |
| | *Bacillus* | *Bacillus mesonae* | 1 |
| | *Bacillus* | *Bacillus pacificus* | 1 |
| | *Bacillus* | *Bacillus wiedmannii* | 2 |
| | *Bacillus* | *Bacillus zanthoxyli* | 1 |
| | *Bacillus* | *Bacillus marisflavi* | 2 |
| | *Bacillus* | *Bacillus cereus* | 12 |
| | *Bacillus* | *Bacillus albus* | 2 |
| | *Bacillus* | *Bacillus aryabhattai* | 6 |
| | *Bacillus* | *Bacillus ginsengisoli* | 1 |
| | *Bacillus* | *Bacillus megaterium* | 10 |
| | *Bacillus* | *Bacillus sp.* | 15 |
| | *Bacillus* | *Bacillus subtilis* | 3 |
| | *Beijerinckia* | *Beijerinckia fluminensis* | 1 |
| | *Chryseobacterium* | *Chryseobacterium culicis* | 4 |
| | *Dyadobacter* | *Dyadobacter endophyticus* | 1 |
| | *Falsibacillus* | *Falsibacillus pallidus* | 18 |
| | *Klebsiella* | *Klebsiella variicola* | 12 |
| | *Lysinibacillus* | *Lysinibacillus sp.* | 1 |
| | *Lysinibacillus* | *Lysinibacillus fusiformis* | 1 |
| | *Microbacterium* | *Microbacterium trichothecenolyticum* | 1 |
| | *Microbacterium* | *Microbacterium laevaniformans* | 1 |
| | *Microbacterium* | *Microbacterium proteolyticum* | 1 |
| | *Oceanobacillus* | *Oceanobacillus picturae* | 2 |
| | ***Paenibacillus*** | *Paenibacillus polymyxa* | 3 |
| | *Peribacillus* | *Peribacillus huizhouensis* | 1 |
| | *Peribacillus* | *Peribacillus simplex* | 1 |
| | *Solibacillus* | *Solibacillus isronensis* | 1 |
| | ***Chryseobacterium*** | *Chryseobacterium culicis* | 1 |
| Alkaline soil | *Aeribacillus* | *Aeribacillus pallidus* | 1 |
| | *Bacillus* | *Bacillus aryabhattai* | 4 |
| | *Bacillus* | *Bacillus asahii* | 12 |
| | *Bacillus* | *Bacillus atrophaeus* | 1 |
| | *Bacillus* | *Bacillus bataviensis* | 4 |
| | *Bacillus* | *Bacillus cereus* | 2 |
| | *Bacillus* | *Bacillus flexus* | 4 |
| | *Bacillus* | *Bacillus ginsengisoli* | 8 |

| | | |
|---|---|---|
| *Bacillus* | *Bacillus haikouensis* | 1 |
| *Bacillus* | *Bacillus megaterium* | 14 |
| *Bacillus* | *Bacillus oryzaecorticis* | 1 |
| *Bacillus* | *Bacillus sp* | 23 |
| *Bacillus* | *Bacillus subtilis* | 1 |
| *Bacillus* | *Bacillus thioparans* | 2 |
| *Bacillus* | *Bacillus velezensis* | 6 |
| *Bacillus* | *Bacillus toyonensis* | 1 |
| *Bacillus* | *Bacillus paraflexus* | 1 |
| *Bacillus* | *Bacillus wiedmannii* | 7 |
| *Bacillus* | *Bacillus zanthoxyli* | 3 |
| *Exiguobacterium* | *Exiguobacterium alkaliphilum* | 1 |
| *Falsibacillus* | *Falsibacillus pallidus* | 1 |
| *Glutamicibacter* | *Glutamicibacter halophytocola* | 1 |
| *Glutamicibacter* | *Glutamicibacter protophormiae* | 1 |
| *Lysinibacillus* | *Lysinibacillus alkaliphilus* | 1 |
| *Lysinibacillus* | *Lysinibacillus fusiformis* | 1 |
| *Lysinibacillus* | *Lysinibacillus xylanilyticus* | 2 |
| *Lysinibacillus* | *Lysinibacillus pakistanensis* | 1 |
| *Lysinibacillus* | *Lysinibacillus macroides* | 1 |
| *Lysinibacillus* | *Lysinibacillus sp.* | 1 |
| *Paenibacillus* | *Paenibacillus polymyxa* | 2 |
| *Peribacillus* | *Peribacillus simplex* | 2 |

**Supplementary Table 3 Agronomic traits of barley plants (WT and *nrh*) under control and *C. culicis* and *P. polymyxa* co-inoculation (+*C*+*P*) in the field.** Values are the mean ± SE (*n* = 6 plots). Significant difference was calculated using one-way ANOVA Tukey's HSD (two-sided).

| Experimental site | Material | Treatments | Plant height (cm) | Spike length (cm) | Grain number spike$^{-1}$ | Filled grain rate (%) | Thousand kernels weight (g) | Grain length (mm) | Grain width (mm) | Harvest index (%) |
|---|---|---|---|---|---|---|---|---|---|---|
| Yangzhou City | WT | Control | 109.7±3.4a | 79.7±3.3a | 21.3±1.5a | 89.0±2.2a | 42.9±1.6a | 10.5±0.2a | 3.5±0.1a | 46.5±1.1a |
| | | +*C*+*P* | 113.7±5.2a | 79.7±1.5a | 22.8±1.0a | 92.4±1.0a | 38.5±0.8a | 10.3±0.3a | 3.4±0.1a | 49.3±1.1a |
| | *nrh* | Control | 107.2±3.0a | 82.5±2.0a | 21.5±1.2a | 90.7±2.0a | 38.5±2.7a | 10.5±0.1a | 3.5±0.1a | 46.1±1.9a |
| | | +*C*+*P* | 110.7±3.9a | 80.3±2.8a | 20.3±1.4a | 94.5±1.2a | 40.2±1.2a | 9.8±0.2a | 3.3±0.1a | 49.0±0.6a |
| Sanming City | WT | Control | 106.5±1.7A | 80.7±2.1A | 23.7±1.5A | 91.3±1.3A | 41.1±1.0A | 10.4±0.1A | 3.5±0.1A | 48.5±1.3A |
| | | +*C*+*P* | 100.5±3.6A | 81.7±1.5A | 23.0±1.3A | 93.7±1.4A | 41.0±0.8A | 10.3±0.2A | 3.5±0.1A | 50.1±1.6A |
| | *nrh* | Control | 102.0±2.6A | 83.5±1.2A | 23.8±1.4A | 91.4±1.4A | 37.5±1.7A | 10.6±0.1A | 3.6±0.1A | 46.7±2.6A |
| | | +*C*+*P* | 102.3±2.9A | 80.5±1.5A | 24.5±1.0A | 94.6±1.3A | 39.6±0.8A | 9.9±0.1A | 3.6±0.1A | 47.1±1.6A |

**Supplementary Table 4 Primers used in this study.**

| Primer name | Primer sequence 5'-3' |
|---|---|
| 27F | AGAGTTTGATCCTGGCTCAG |
| 1492R | GGTTACCTTGTTACGACTT |
| pRE112-*trpC*_UP_F | ATCGCATGCGGTACCTCTAGGAAATAAGCCTTACCGACG |
| pRE112-*trpC*_UP_R | TGCAGGTTCATTGATACAGAATTTAATAAGATGAT |
| pRE112-*trpC*_DOWN_F | TCTGTATCAATGAACCTGCAACCACAAC |
| pRE112-*trpC*_DOWN_R | CGATCCCAAGCTTCTTCTAGAGTAGGGAATGGGAATCTGG |
| *trpC*_Check_F | ATCGCATGCGGTACCTCTAGGAAATAAGCCTTACCGACG |
| *trpC*_Check_R | CGATCCCAAGCTTCTTCTAGAGTAGGGAATGGGAATCTGG |
| pRE112-*ipdC*_UP_F | ATCGCATGCGGTACCTCTAGAACGGGCCTCCTTTTCTACC |
| pRE112-*ipdC*_UP_R | TGTTGTCATTTGGTCATCCTCCTTTACAAAATT |
| pRE112-*ipdC*_DOWN_F | AGGATGACCAAATGACAACAAAAAATACCATTGCT |
| pRE112-*ipdC*_DOWN_R | CGATCCCAAGCTTCTTCTAGTTCGAGCCCCCGCTCTTA |
| *ipdC*_Check_F | ATCGCATGCGGTACCTCTAGAACGGGCCTCCTTTTCTACC |
| *ipdC*_Check_R | CGATCCCAAGCTTCTTCTAGTTCGAGCCCCCGCTCTTA |

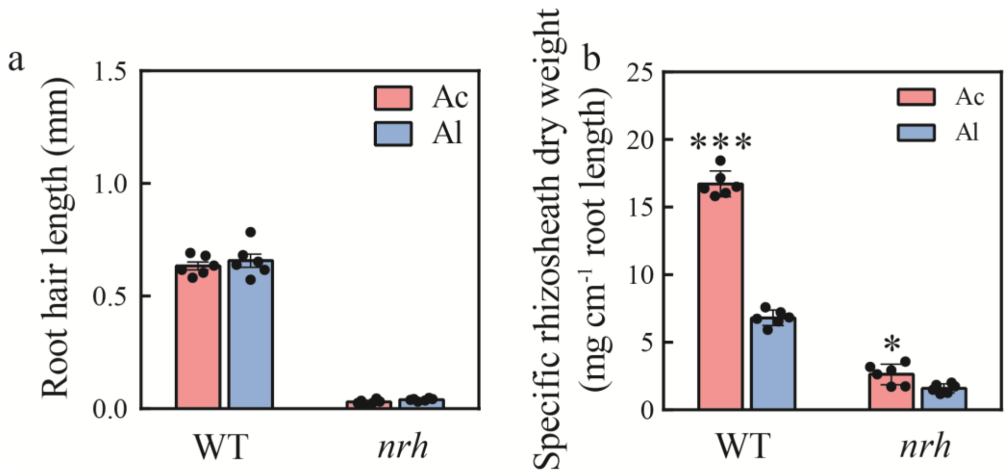


**Supplementary Fig. 1 Barley rhizosheath formation is also increased in acid soil under control condition (WW: well water).** a, Root hair length of WT and *nrh* in Ac and Al soils under WW. b, The specific rhizosheath soil dry weight of WT and *nrh* in Ac and Al soils under WW. Data are means ± SE ($n$ = 6 independent replicates). Asterisks indicate significant difference by two-sided Student's $t$-test (*$P < 0.05$, *** $P < 0.001$). The exact $p$ values are provided in the Source Data file.

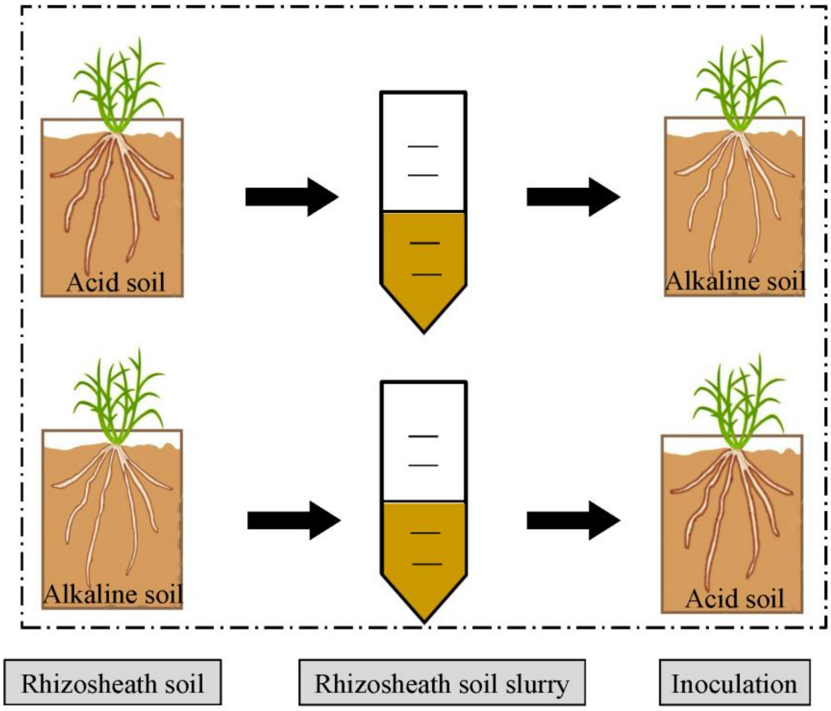


**Supplementary Fig. 2 Schematic representation of the transplantation experiment between two soil types.**

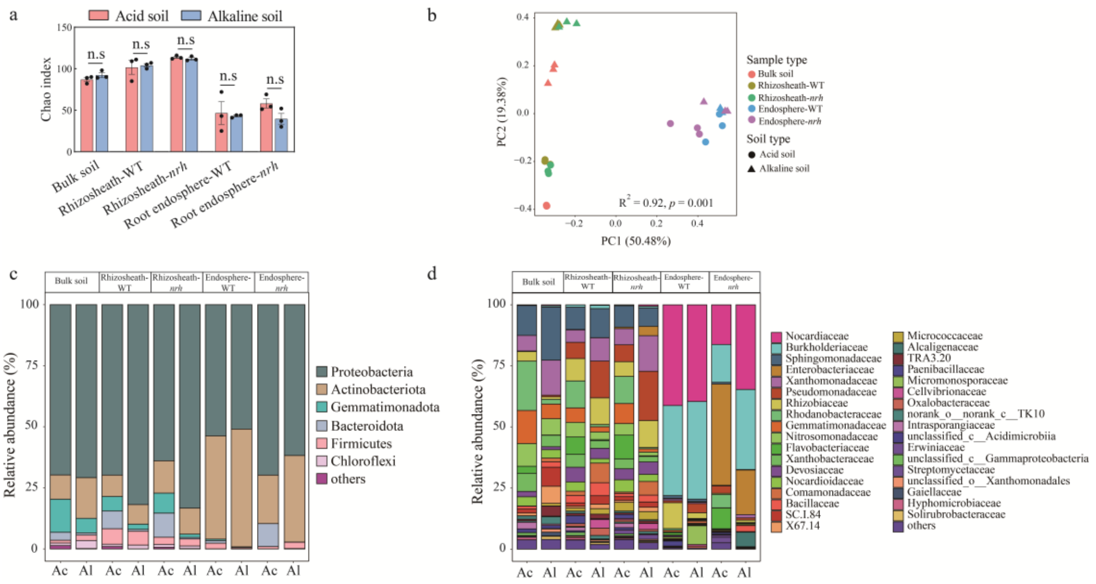


**Supplementary Fig. 3 Bacterial community of WT and *nrh* in acid and alkaline soils under WW conditions. a**, Estimated Chao indexe in acid and alkaline soils under WW conditions. Data are means ± SE ($n$ = 3 independent replicates). Significant difference was calculated using the two-sided Student's $t$-test. **b,** Principal-coordinate analysis (PCoA) analysis based on Bray-Curtis distance in the bulk soil, rhizosheath and root endosphere of WT and *nrh* barley plants in acid soil and alkaline soil under WW conditions ($p$ = 0.001; PERMANOVE by Adonis test). **c-d,** The phylum level and family level relative abundance in bulk soil, rhizosheath and root endosphere samples in acid soil and alkaline soil under WW conditions.

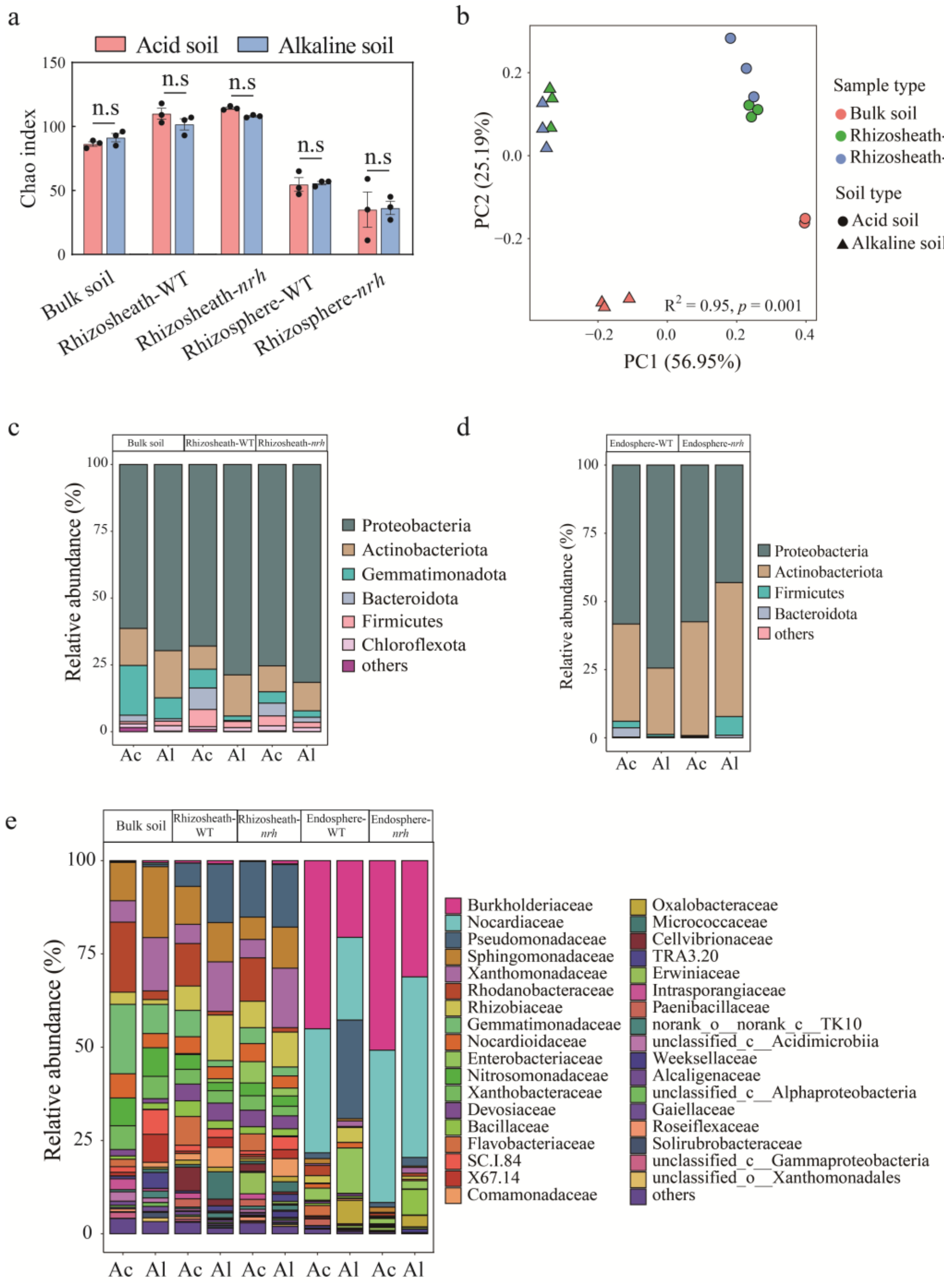


**Supplementary Fig. 4 Bacterial community of WT and *nrh* in acid and alkaline soils under MSD conditions. a**, Estimated Chao indexe in acid and alkaline soils under MSD conditions. Data are means ± SE (*n* = 3 independent replicates). Significant difference was calculated using the two-sided Student's *t*-test. **b,** Principal co-ordinate analysis (PCoA) based on Bray-Curtis distance in the bulk soil and rhizosheath of WT and *nrh* plants in acid and alkaline soils under MSD ($p$ = 0.001; PERMANOVE by Adonis test). **c**, Phylum relative abundances of bulk soil and rhizosheath samples in acid and alkaline soils under MSD. **d,** The phylum level relative abundance in root endosphere samples in acid soil and alkaline soil under

MSD conditions. **e,** The family level relative abundance in bulk soil, rhizosheath and root endosphere samples in acid soil and alkaline soil under MSD conditions.

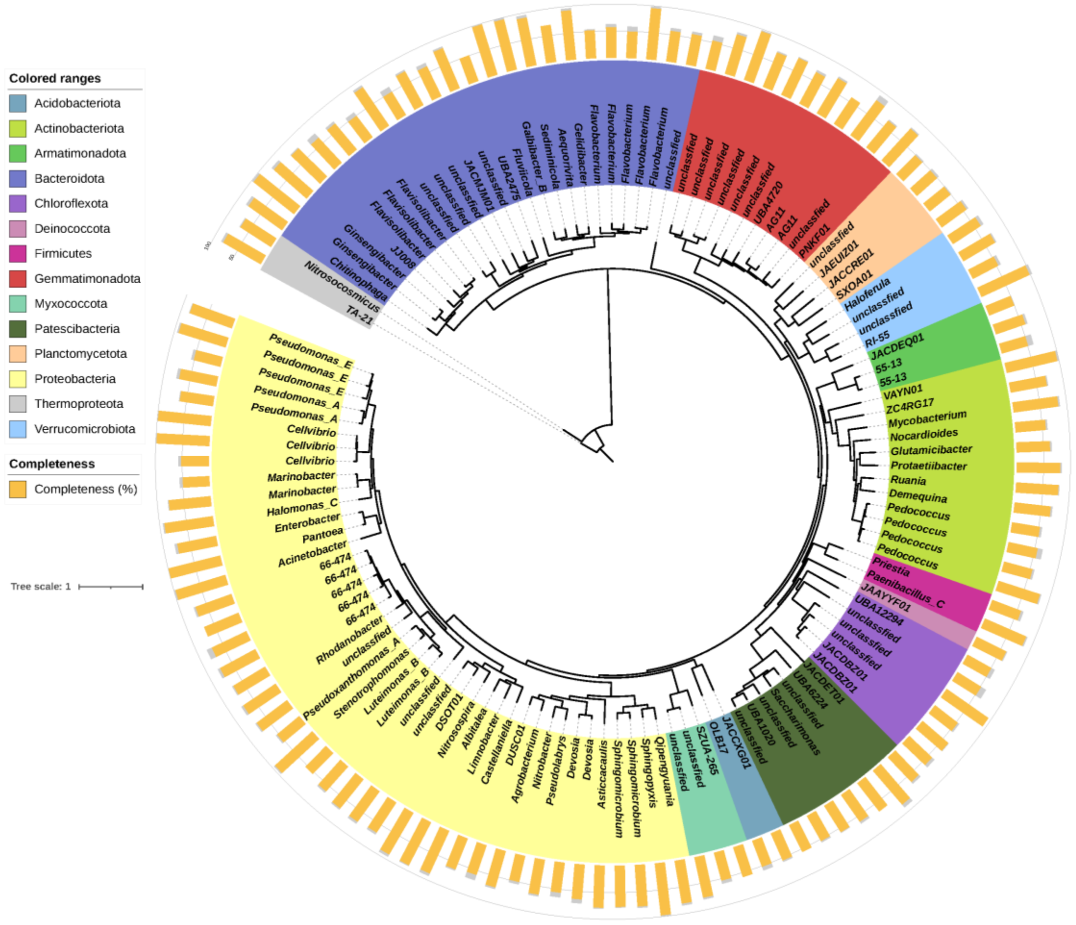


**Supplementary Fig. 5** A phylogenetic tree of 124 bacterial MAGs recovered from metagenomes of all samples. Inner circles are colored by phylum-level taxonomy. Yellow bar indicates the completencess (%) in MAGs. Tree scale shows nucleotide substitutions for each site.

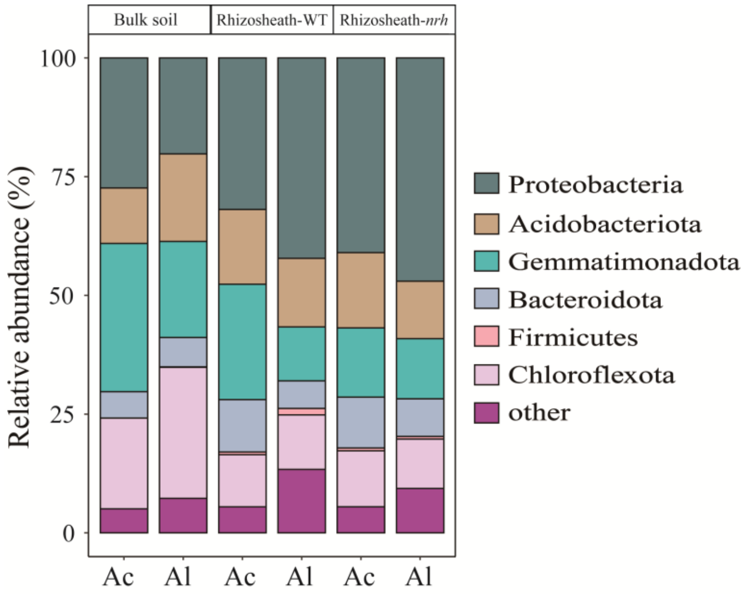


**Supplementary Fig. 6** Comparison of taxonomic composition and relative bacterial abundances at phylum level based on 124 MAGs of WT and *nrh* plants in acid and alkaline soils under MSD.

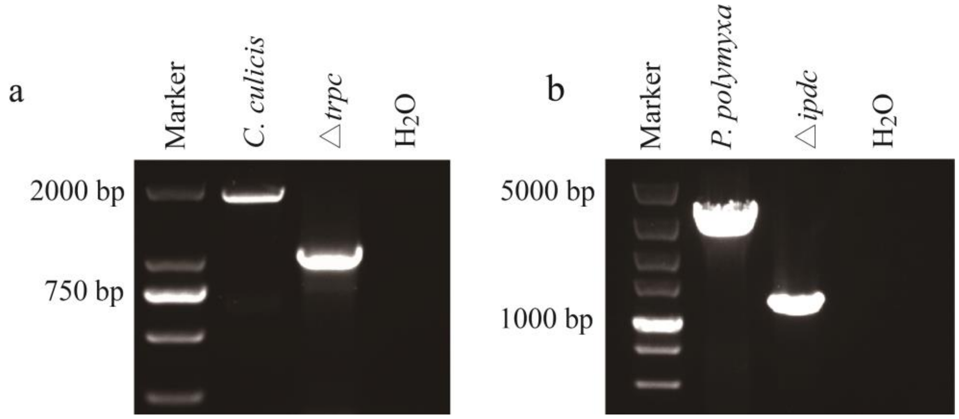


**Supplementary Fig. 7 a-b,** Identification of IAA biosynthesis mutant △*trpc* (a) and △*ipdc* (b) in the genetic background of *C. culicis*.

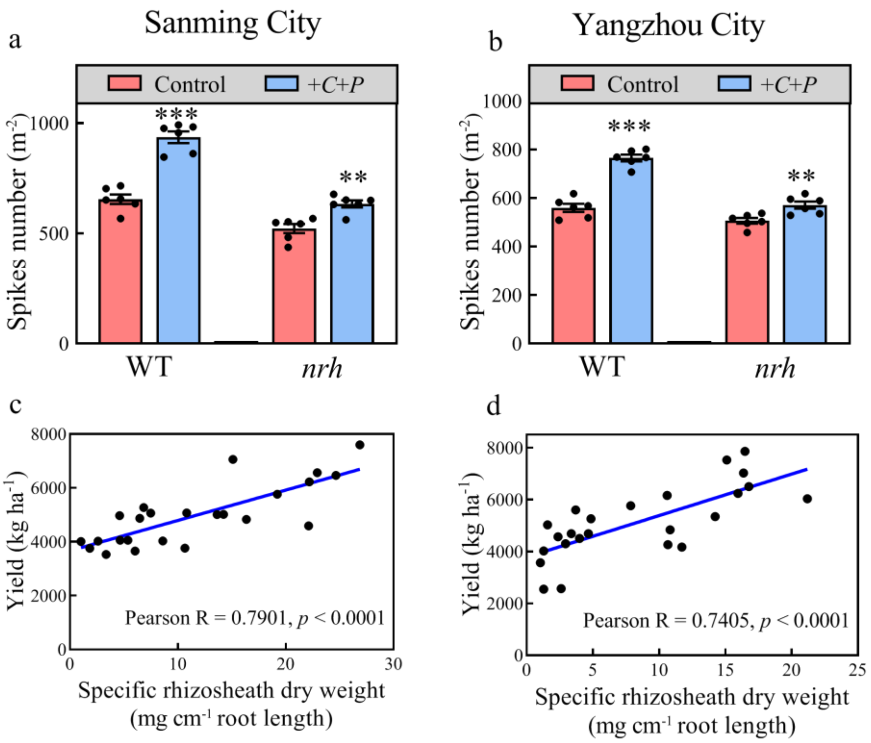


**Supplementary Fig. 8 a-b, Spikes number of barely at two locations.** Data are means ± SE ($n$ = 6 plots) (a, b). Asterisks indicate significant difference between different treatments by two-sided Student's $t$ test (a, b; *$p < 0.05$, ** $p < 0.01$, *** $p < 0.001$). **c-d, Correlation between rhizosheath formation and grain yield of barely at two locations.** $p$ values was calculated based on two-sided $t$ test. The exact $p$ values are provided in the Source Data file.

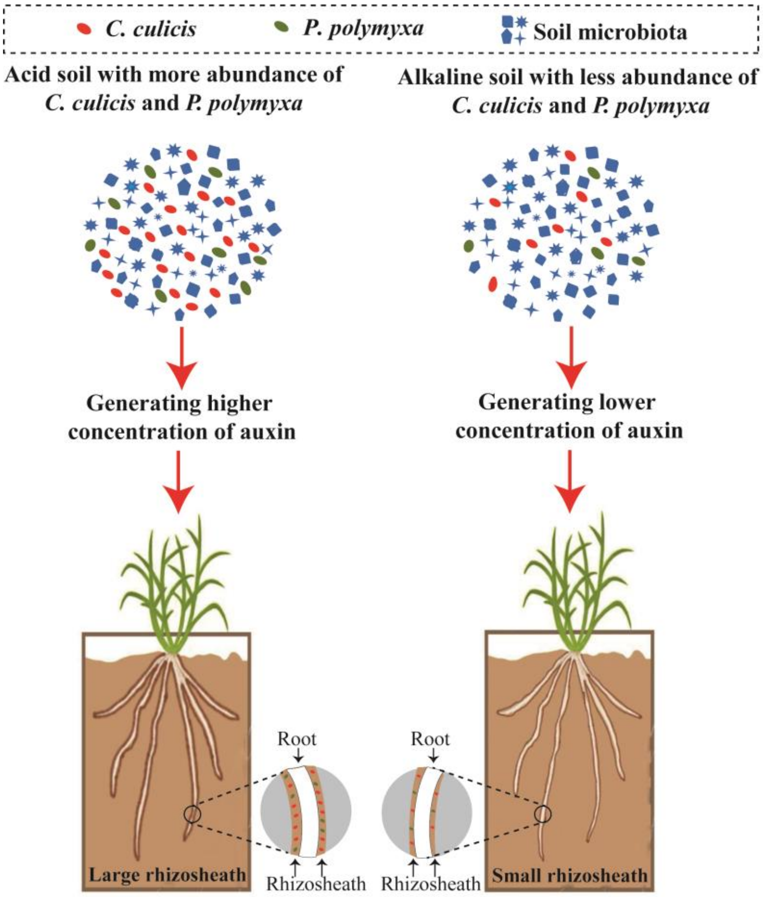


**Supplementary Fig. 9 Soil bacterial communities contribute to barley rhizosheath formation in acid soil or alkaline soil.** Briefly, *C. culicis* and *P. polymyxa* contribute to barley root hair growth through IAA production, thereby increasing barley rhizosheath formation in acid or alkaline soil.
