# Supplementary material for: Auxin-producing bacteria promote barley rhizosheath formation
Source: Nat Commun. 2023 Sep 19;14:5800. doi: 10.1038/s41467-023-40916-4 (PMC10509245; doi:10.1038/s41467-023-40916-4)
Supplement: Supplementary file 3 — Description of Additional Supplementary Files [file 41467_2023_40916_MOESM3_ESM.pdf]

Description of Additional Supplementary Files:

Supplementary Data 1: Mean estimated bacterial taxa and changes at phylum and family levels.

Supplementary Data 2: Relative abundance of the Flavobacteriaceae and Paenibacillaceae of WT and nrh plants in acid and alkaline soils from metagenomic analysis.

Supplementary Data 3: IAA biosynthesis related KEGG pathways of WT and nrh plants in acid and alkaline soils from metagenomic anaysis.

Supplementary Data 4: IAA biosynthesis related genes in key bins from metagenomic anaysis.

Supplementary Data 5: Possbile IAA biosynthesis related genes in C. culicis.

Supplementary Data 6: Possbile IAA biosynthesis related genes in P. polymyxa.

Supplementary Data 7: Verification of trpC and ipdC deletion strain using DNA sequencing.
