# Supplementary material for: Auxin-producing bacteria promote barley rhizosheath formation
Source: Nat Commun. 2023 Sep 19;14:5800. doi: 10.1038/s41467-023-40916-4 (PMC10509245; doi:10.1038/s41467-023-40916-4)
Supplement: Supplementary file 5 — Supplementary Dataset 7 [file 41467_2023_40916_MOESM5_ESM.zip › Supplementary Data 7.docx]

**Supplementary Data 7** Verification of *trpC* and *ipdC* deletion strain using DNA sequencing. **A,** Verification of *trpC* deletion strain using DNA sequencing. The highlighted sequence is the coding sequence (CDS) region of *trpC*. The green highlighted sequence is the knockdown region in mutant strain. B, Verification of *ipdC* deletion strain using DNA sequencing. The highlighted sequence is the coding sequence (CDS) region of *ipdC*. The green highlighted sequence is the knockdown region in mutant strain. D, DNA mutation of *ipdC* gene in △*ipdc*. The same sequence is displayed in blue.

**A**

>***C. culicis_TrpC***

GAAATAAGCCTTACCGACGACAGCAAAATCATCACAAAAAATGGTGAAGAAATCTATTCTGCGGAAGATTTAGGCTTCAATCCTGTAACATTGGAAGATATTAAAGCAGGAAGTTCTATTCAGGAAACAGCAAAAATATTTATGAATATTCTGGAAGGAAAAGGTACAGAACAACAGAACGCTGTCATTCTTGCCAATGCTTCAGTAGCCCTTTACAACACTCATAAATTCGGGACATATGAAGACTGTCTTCTGCTGGCTAAAGAAAGTTTACAAAGTGGAAAAGCATTGAATAGTTTTAATCTTTTAATTCATTAGTTTTATTATTTTTTTTAAACCGTTAAGAATTTTTAAGTGATCAAGAGTAGTTAAGATCATAGCTAAAGCTATTTATTTAGCGACATCCTTCATTCTCCATCTTGATGGAACTTAATTTATCTTATCATCTTAATATCCCTTAATGGTTTATCATTTTTATCATCTTATTAAATTCTGTATCAAATGACCATACTGGATAAAATTATTGAACGAAAAAAAGAGGAAGTTGCTGGAGCAAAGTCACGTATTTCTATTGACCGATTAAAGAACACTGCTTTTTTTGGAAGAAAAACCCATTCCCTGAAAGAATCCATCAAAAATAAAAGCGGAATTATCGCTGAATTTAAAAGACAATCACCATCCAAAGGGATCATCAACAATAGTGTTGAACCTTTAGATGTTGTTTCAGCCTATGAAAGTTTTGGAGCCAGTGGAATTTCTATCCTGACAGATCATGACTTCTTCGGAGGAAATTTAAATGATGTTGTAAGTGTAAGAAATGAGATTAATATTCCGATTCTACGAAAAGATTTCATGATTGATGAATATCAGTTTTATGAAGCTAAAAGCATTGGTGCTGATGTTGTTCTGTTGATTGCTTCATGTCTTTCTCCTGCGCAGGTACAAGAATTTACAGAACTCGCTCATGAACTGAAATTGGAAGTTTTGCTGGAAATTCATACAGAAGACGAACTAAAGTATTTCAATTCAGAAATCGACTTGGTAGGGATCAATAACAGGAATCTTAAAGATTTTAAAGTAGATCTGCAGCATTCTGTTCAGTTGAAAAATCAGCTTCCGAAAGGAGTTTTATCCGTTGCAGAAAGCGGTATTTACAGCCTTGAAGACTTTATATTTTTAAAAGAAAAAGGATTTGACGGCTTCCTGATGGGAGAATATTTCATGAAAAACACCAATCCTGCCAAATCTTTTGAAGAATTTTCTTCTCAAATTTATAATCAATCATAATGAACCTGCAACCACAACTAAAAGTCTGCGGTCTTACAAAAACAGACCAGATTCAGGAACTAATTTCTATGGATGTAGATTTTCTTGGTTTCATCTTCTATGAAAAATCACCCAGATATGTTTTGAATCATCTGAGTCTGGAAGAAATCTCGACGATTGATCATCATGGAAAAGTAGGTGTTTTTGTGAATGAAGAAATGGACAAAATTGTAAGTATTGTTCAACAGGCGGATTTGAATTTCGTTCAGCTTCATGGTGATGAAAATAATGATTTTATTACTGAATTAAGACAAAAACTGTATCCGGAAGTTGGTATTATTAAAGTAATCAGAATAGGAAATATTGATTCTGATAATAAAGATAAAATAACCCAAACATTTACCTCCAATCTGCAACCTGTCACCTATTATCTCTTTGATACTGACAGTAAAGCCTTTGGAGGAACGGGAAAACAATTCGACTGGAATATCCTGAATGAATTCCAGATTCCCATTCCCTACT

>***C. culicis_*△*trpc***

GAAATAAGCCTTACCGACGACAGCAAAATCATCACAAAAAATGGTGAAGAAATCTATTCTGCGGAAGATTTAGGCTTCAATCCTGTAACATTGGAAGATATTAAAGCAGGAAGTTCTATTCAGGAAACAGCAAAAATATTTATGAATATTCTGGAAGGAAAAGGTACAGAACAACAGAACGCTGTCATTCTTGCCAATGCTTCAGTAGCCCTTTACAACACTCATAAATTCGGGACATATGAAGACTGTCTTCTGCTGGCTAAAGAAAGTTTACAAAGTGGAAAAGCATTGAATAGTTTTAATCTTTTAATTCATTAGTTTTATTATTTTTTTTAAACCGTTAAGAATTTTTAAGTGATCAAGAGTAGTTAAGATCATAGCTAAAGCTATTTATTTAGCGACATCCTTCATTCTCCATCTTGATGGAACTTAATTTATCTTATCATCTTAATATCCCTTAATGGTTTATCATTTTTATCATCTTATTAAATTCTGTATCAATGAACCTGCAACCACAACTAAAAGTCTGCGGTCTTACAAAAACAGACCAGATTCAGGAACTAATTTCTATGGATGTAGATTTTCTTGGTTTCATCTTCTATGAAAAATCACCCAGATATGTTTTGAATCATCTGAGTCTGGAAGAAATCTCGACGATTGATCATCATGGAAAAGTAGGTGTTTTTGTGAATGAAGAAATGGACAAAATTGTAAGTATTGTTCAACAGGCGGATTTGAATTTCGTTCAGCTTCATGGTGATGAAAATAATGATTTTATTACTGAATTAAGACAAAAACTGTATCCGGAAGTTGGTATTATTAAAGTAATCAGAATAGGAAATATTGATTCTGATAATAAAGATAAAATAACCCAAACATTTACCTCCAATCTGCAACCTGTCACCTATTATCTCTTTGATACTGACAGTAAAGCCTTTGGAGGAACGGGAAAACAATTCGACTGGAATATCCTGAATGAATTCCAGATTCCCATTCCCTACT

**B**

>***P. polymyxa_ipdC***

AACGGGCCTCCTTTTCTACCCTTTTAACAAGATAAGACTTGGCTTTGGAGGAGTGCACTGTCTCACCGTGTGGTCTTTTTTATAATATGCGGGTACCTCTTACAAGCGTGCGACTCTGAGGTGCTTCAGAAAAGAAACATAAATTTTTATGAAATATGGGTATTGACTATCCCAGATGGGAGACATATAATGTGTCTAAACATTTGAAAGAATGACATGAACTCAACGGCAATGAAGAGGACGAAGTGATTAGGGCTCTTTTGTTCAGAGAGTGAGGCATTAGCTGCAAGCTTCACCAAAATCCCTTTTTAACGAGCTCACCTTGGAGCTGTTTTCCTGAAAAGTATTTGGGCCCGATGATATTAACGGTGCGCTGAATCACCTATTAGGGAAAATCGTACGGTCTACGTTACAGACGCCAGATATGGAGATCCTTTGCGATTTCTGTACCTGCCAAGGTTCGATATTGCGAAATATCGGGCAAACACGGGTGGTACCACGGAAGCTACAGCCTTTCGTCCCTCAGTAACAGCAAGTCTGTTCTAGGGATGAAAGGTTTTTTTGTTTTTACAAATGTCAGATGTATGTAAATTTTGTAAAGGAGGATGACCAATGAGTGCACAAATTCCTGAAGTTAGGTCAACCAATGAATTACGTGAAAAGTGGATGAAGCCTGAAGTGATTACAGGTTCGGAAATTTTACTCCGTAGCCTGTTGCTGGAGGGAGTAGATTGTGTCTTCGGATATCCTGGTGGTGCAGTGCTTTATATCTATGATGCCATGTATGGCTTTAAGGATTTCAAGCATGTCCTCACTCGTCACGAGCAGGGAGCTATTCATGCGGCAGATGGTTATGCACGGGCGAGCGGTAAAGTGGGCGTATGTATCGCGACTTCCGGACCTGGAGCGACAAACCTGGTAACTGGTATTGCGACGGCTTTTATGGATTCGGTGCCGTTGGTGGTCATCACGGGTAACGTTATATCTTCCCTGATCGGTACGGATGCTTTCCAGGAAGCCGATATCACGGGAATTACAATGCCGATTACGAAGCACAGCTATCTGGTAAGAGATGTAGAGGATCTGCCGCGAATCATCCATGAGGCATTTCATATTGCCAACACAGGGCGGAAGGGACCGGTGCTGATTGATATTCCGAAGGATATTTCCGCAGCCCAGACGTTGTTCGTTCCACAAACGAGACCAGTGACCATGCGGGGTTATAACCCGAAAGTGCTGCCCAACAAAATTCAACTAGACAAGCTAGCTCAAGCTATTTCCGAAGCAGAACGTCCATTCATTCTGGCAGGTGGAGGAGTTGTATACTCCGGTGGACATGAAGCACTTTACGAGTTTGTTCGTAAAACGGAAATTCCTATTACGACAACATTGCTCGGATTGGGTGGTTTCCCAAGTGGACATGAGCTGTGGACGGGGATGCCGGGGATGCATGGAACATACACCTCCAATCAGGCGATTCAACAGTCAGATCTGTTGATCTGTATCGGAGCCCGCTTCGATGATCGGGTAACAGGCAAGTTGGATGGGTTTGCTCCTCAAGCTAAAATCGTCCACATTGATATCGACCCTGCTGAAATTGGAAAAAATGTTGCAGCAGATATTCCTATCGTGGGTGACGTGAAGGCAGTGCTGGAATTGCTGAATCAGGATGTGAAACGCGCGGATCGAGCTGATGCATGGAGAGCACAAATTCAGCATTGGAAGAATGAGAAACCTTATTCTTATAAGGATTCTGAAACGGTGCTTAAACCACAATGGGTTGTTGAGTTGTTGGATGAAACGACCAAGGGCGGCGCAATCGTAACGACGGACGTGGGACAGCATCAAATGTGGGCAGCGCAGTATTACAAATTCAATCAGCCACGCTCATGGGTCACCTCTGGTGGGCTCGGTACGATGGGATTTGGTTTCCCCTCTGCAATCGGTGCTCAGATGGCCAATCCCGACAGATTAGTAATATCGATTAACGGGGACGGCGGTATGCAGATGTGTTCGCAGGAATTAGCCATCTGTGCAATCAATAACATTCCGGTTAAAATCGTAATTATCAACAACCAGGTGCTTGGAATGGTTCGCCAATGGCAGGAATTGATCTATAACAATCGGTATAGTCACATTGACCTGGCTGGAAGTCCGGATTTTGTGAAACTTGCCGAAGCATACGGTGTCAAAGGACTGCGTGCTACGAATAAGGAAGAGGCACGTCGGGCTTGGCAGGAGGCGCTTGATACACCCGGACCGGTCGTTGTCGAGTTTGTAGTCAGCAAGGAAGAGAATGTATATCCGATGGTGACGCAAGGTTCGACAATTGATCAAATGCTGATGGGGGACGAGTAAAATGACAACAAAAAATACCATTGCTGTACTGGTAAATGATCAACCCGGCGTTTTGCAACGTGTGTCCGGCCTGTTCGGTCGCCGAGGCTTTAACATTGAGAGCATAACTGTGGGACAATCGGAAGAAGTTGGATTATCTCGTATGGTCATTGTGACTGTAGGGGATGAGAACAACCTGGAGCAGATTGAAAAGCAGCTTTATAAGCTGGTTGATGTGATCAAGGTCATTGATCTCAGTTCCAAGCCGATGGTAGCTCGGGAATTGGCGATGATAAAGGTAAAGGCAGAACCACCTCAGCGGCCAGAGATCATGGGCGTGGTAGAAACATTCAGAGCAGCTGTAATTGATGTTGGAACGACGAGCTTGATCGTACAGGTCATTGGCGATACAGAAAAAATTGATGCCATGATCGAGTTGTTGAAGCCTTACGGTATTCGGGAGTTGACTCGAACCGGGGTTACAGCTATGATACGTGGCAATGCTTAATATGGAGCTTTAACGAATTACCGCTTATAGCGCATTACATCAATAAAGAACCGCCCGCATAAGAGCGGGGGCTCGAA

> ***P. polymyxa_*△*ipdc***

AACGGGCCTCCTTTTCTACCCTTTTAACAAGATAAGACTTGGCTTTGGAGGAGTGCACTGTCTCACCGTGTGGTCTTTTTTATAATATGCGGGTACCTCTTACAAGCGTGCGACTCTGAGGTGCTTCAGAAAAGAAACATAAATTTTTATGAAATATGGGTATTGACTATCCCAGATGGGAGACATATAATGTGTCTAAACATTTGAAAGAATGACATGAACTCAACGGCAATGAAGAGGACGAAGTGATTAGGGCTCTTTTGTTCAGAGAGTGAGGCATTAGCTGCAAGCTTCACCAAAATCCCTTTTTAACGAGCTCACCTTGGAGCTGTTTTCCTGAAAAGTATTTGGGCCCGATGATATTAACGGTGCGCTGAATCACCTATTAGGGAAAATCGTACGGTCTACGTTACAGACGCCAGATATGGAGATCCTTTGCGATTTCTGTACCTGCCAAGGTTCGATATTGCGAAATATCGGGCAAACACGGGTGGTACCACGGAAGCTACAGCCTTTCGTCCCTCAGTAACAGCAAGTCTGTTCTAGGGATGAAAGGTTTTTTTGTTTTTACAAATGTCAGATGTATGTAAATTTTGTAAAGGAGGATGACCAAATGACAACAAAAAATACCATTGCTGTACTGGTAAATGATCAACCCGGCGTTTTGCAACGTGTGTCCGGCCTGTTCGGTCGCCGAGGCTTTAACATTGAGAGCATAACTGTGGGACAATCGGAAGAAGTTGGATTATCTCGTATGGTCATTGTGACTGTAGGGGATGAGAACAACCTGGAGCAGATTGAAAAGCAGCTTTATAAGCTGGTTGATGTGATCAAGGTCATTGATCTCAGTTCCAAGCCGATGGTAGCTCGGGAATTGGCGATGATAAAGGTAAAGGCAGAACCACCTCAGCGGCCAGAGATCATGGGCGTGGTAGAAACATTCAGAGCAGCTGTAATTGATGTTGGAACGACGAGCTTGATCGTACAGGTCATTGGCGATACAGAAAAAATTGATGCCATGATCGAGTTGTTGAAGCCTTACGGTATTCGGGAGTTGACTCGAACCGGGGTTACAGCTATGATACGTGGCAATGCTTAATATGGAGCTTTAACGAATTACCGCTTATAGCGCATTACATCAATAAAGAACCGCCCGCATAAGAGCGGGGGCTCGAA
