# Supplementary material for: Auxin-producing bacteria promote barley rhizosheath formation
Source: Nat Commun. 2023 Sep 19;14:5800. doi: 10.1038/s41467-023-40916-4 (PMC10509245; doi:10.1038/s41467-023-40916-4)
Supplement: Supplementary file 6 — Reporting Summary [file 41467_2023_40916_MOESM6_ESM.pdf]

nature portfolio

Corresponding author(s): Weifeng Xu

Last updated by author(s): Aug 9, 2023

Reporting Summary

Nature Portfolio wishes to improve the reproducibility of the work that we publish. This form provides structure for consistency and transparency in reporting. For further information on Nature Portfolio policies, see our Editorial Policies and the Editorial Policy Checklist.

Statistics

For all statistical analyses, confirm that the following items are present in the figure legend, table legend, main text, or Methods section.

n/a	Confirmed	
☐	☒	The exact sample size (*n*) for each experimental group/condition, given as a discrete number and unit of measurement
☐	☒	A statement on whether measurements were taken from distinct samples or whether the same sample was measured repeatedly
☐	☒	The statistical test(s) used AND whether they are one- or two-sided<br>*Only common tests should be described solely by name; describe more complex techniques in the Methods section.*
☐	☒	A description of all covariates tested
☐	☒	A description of any assumptions or corrections, such as tests of normality and adjustment for multiple comparisons
☐	☒	A full description of the statistical parameters including central tendency (e.g. means) or other basic estimates (e.g. regression coefficient) AND variation (e.g. standard deviation) or associated estimates of uncertainty (e.g. confidence intervals)
☐	☒	For null hypothesis testing, the test statistic (e.g. *F*, *t*, *r*) with confidence intervals, effect sizes, degrees of freedom and *P* value noted<br>*Give P values as exact values whenever suitable.*
☒	☐	For Bayesian analysis, information on the choice of priors and Markov chain Monte Carlo settings
☒	☐	For hierarchical and complex designs, identification of the appropriate level for tests and full reporting of outcomes
☐	☒	Estimates of effect sizes (e.g. Cohen's *d*, Pearson's *r*), indicating how they were calculated

Our web collection on statistics for biologists contains articles on many of the points above.

Software and code

Policy information about availability of computer code

Data collection	Image J (v1.8.0); NovaSeq6000 platform
Data analysis	All codes used in the study are available at GitHub (https://github.com/xufychallenge/xufy-NC.git).<br><br>QIIME2; DADA2; SILVA (v 138); Trimmomatic (v 0.39); Bowtie2 v 2.5.0; SPAdes v 3.13.1; MetaWRAP; MetaBAT2; MaxBin2; Concoct; CheckM (v 1.0.13); dRep v 2.3.2; GTDB-Tk toolkit (v 0.3.2); CoverM pipeline63 (v 0.61); Prodigal (v 2.6.3); CD-HIT (v 4.8.1); BBMap (v 38.90); KEGG (Release 101.0); eggNOG 5.0; DIAMOND; TBtools (v 1.120); SortMeRNA (v 4.3.4); TopHat 2 (v 2.1.1); Minimap2; HISAT2; GraphPad Prism 7.0; iTOL. V6.4; SPSS v. 20.0; R v4.0.0

For manuscripts utilizing custom algorithms or software that are central to the research but not yet described in published literature, software must be made available to editors and reviewers. We strongly encourage code deposition in a community repository (e.g. GitHub). See the Nature Portfolio guidelines for submitting code & software for further information.

Data

Policy information about availability of data

All manuscripts must include a data availability statement. This statement should provide the following information, where applicable:

- Accession codes, unique identifiers, or web links for publicly available datasets
- A description of any restrictions on data availability
- For clinical datasets or third party data, please ensure that the statement adheres to our policy

16S rRNA gene amplicon sequencing data for the study have been uploaded to the NCBI SRA (https://www.ncbi.nlm.nih.gov) under accession number: PRJNA867556 (https://www.ncbi.nlm.nih.gov/search/all/?term=PRJNA867556). The metagenome and metatranscriptome sequencing data are deposited in Genome Sequence Archive (GSA, https://ngdc.cncb.ac.cn/) in the BIG Data Center, Chinese Academy of Science under BioProject accessions PRJCA016632 and PRJCA016646. The genome of P. polymyxa is deposited in NCBI under BioProject accessions PRJNA908138. The genome of C. culicis is deposited in GSA under BioProject accessions PRJCA016210 (https://ngdc.cncb.ac.cn/search/?dbId=&q=PRJCA016210).

Human research participants

Policy information about studies involving human research participants and Sex and Gender in Research.

Reporting on sex and gender	N/A
Population characteristics	N/A
Recruitment	N/A
Ethics oversight	N/A

Note that full information on the approval of the study protocol must also be provided in the manuscript.

Field-specific reporting

Please select the one below that is the best fit for your research. If you are not sure, read the appropriate sections before making your selection.

☒ Life sciences ☐ Behavioural & social sciences ☐ Ecological, evolutionary & environmental sciences

For a reference copy of the document with all sections, see nature.com/documents/nr-reporting-summary-flat.pdf

Life sciences study design

All studies must disclose on these points even when the disclosure is negative.

Sample size	For experiments in this study involving rhizosheath and soil samples collected for sequencing , three replicates were chosen as in our prior publication for microbiome analysis (See citations in the manuscript for Xu et al., 2022, ISME Journal). We performed statistical tests with obtained data to ensure that a sample size with six independent replicates is sufficient to draw conclusions about significance.
Data exclusions	No data were excluded from the study.
Replication	The validation experiments such as transplantation and inoculation experiments were performed twice in three month. The microbiome sequencing were performed once with three independent replicates.
Randomization	Position of boxes with planted pots was randomized in the greenhouse during the course of the experiments.
Blinding	For all plant experiments, the treatments were assigned a number prior to inoculation and plates were labelled with number and letter codes to prevent potential biases due to knowledge of treatment information during data collection. Metadata was then added before analysis.

Reporting for specific materials, systems and methods

We require information from authors about some types of materials, experimental systems and methods used in many studies. Here, indicate whether each material, system or method listed is relevant to your study. If you are not sure if a list item applies to your research, read the appropriate section before selecting a response.

Materials & experimental systems

n/a	Involved in the study
☒	☐ Antibodies
☒	☐ Eukaryotic cell lines
☒	☐ Palaeontology and archaeology
☒	☐ Animals and other organisms
☒	☐ Clinical data
☒	☐ Dual use research of concern

Methods

n/a	Involved in the study
☒	☐ ChIP-seq
☒	☐ Flow cytometry
☒	☐ MRI-based neuroimaging
