# Supplementary material for: Auxin-producing bacteria promote barley rhizosheath formation
Source: Nat Commun. 2023 Sep 19;14:5800. doi: 10.1038/s41467-023-40916-4 (PMC10509245; doi:10.1038/s41467-023-40916-4)
Supplement: Supplementary file 7 — Source Data [file 41467_2023_40916_MOESM7_ESM.zip › Source Data/p-values.docx]

1. The exact *p* values in **FIG.1** are 0.012 (AC-WT vs. AL-WT) under natural soil, 0.81 (AC-nrh vs. AL-nrh) under natural soil, 0.035 (AC-WT vs. AL-WT) under sterilized soil, 0.769 (AC-nrh vs. AL-nrh) under sterilized soil for **E**; 2.3×10^−8^ (AC-WT vs. AL-WT) under natural soil, 1.6×10^−5^ (AC-nrh vs. AL-nrh) under natural soil, 1×10^−5^ (AC-WT vs. AL-WT) under sterilized soil, 0.977 (AC-nrh vs. AL-nrh) under sterilized soil for **F**; 0.267 (Al-Sterilized vs. Al-Natural) in acid soil, 0.0016 (Ac-Sterilized vs. Ac-Natural) in alkaline soil for **G**; 0.516 (Al-Sterilized vs. Al-Natural) in acid soil, 0.0099 (Ac-Sterilized vs. Ac-Natural) in alkaline soil for **H**.

3. **Fig. 3C-acid soil**

| I | J | p values |
| --- | --- | --- |
| MSD | MSD+C+P | 0.000 |
|  | MSD+NPA | 0.000 |
|  | MSD+C+P+NPA | 0.001 |
| MSD+C+P | MSD | 0.000 |
|  | MSD+NPA | 0.000 |
|  | MSD+C+P+NPA | 0.000 |
| MSD+NPA | MSD | 0.000 |
|  | MSD+C+P | 0.000 |
|  | MSD+C+P+NPA | 0.000 |
| MSD+C+P+NPA | MSD | 0.001 |
|  | MSD+C+P | 0.000 |
|  | MSD+NPA | 0.000 |

**Fig. 3C-alkaline soil**

| I | J | p values |
| --- | --- | --- |
| MSD | MSD+C+P | 0.000 |
|  | MSD+NPA | 0.000 |
|  | MSD+C+P+NPA | 0.044 |
| MSD+C+P | MSD | 0.000 |
|  | MSD+NPA | 0.000 |
|  | MSD+C+P+NPA | 0.000 |
| MSD+NPA | MSD | 0.000 |
|  | MSD+C+P | 0.000 |
|  | MSD+C+P+NPA | 0.000 |
| MSD+C+P+NPA | MSD | 0.044 |
|  | MSD+C+P | 0.000 |
|  | MSD+NPA | 0.000 |

**Fig. 3D-acid soil**

| I | J | p values |
| --- | --- | --- |
| MSD | MSD+C+P | 0.003 |
|  | MSD+NPA | 0.001 |
|  | MSD+C+P+NPA | 0.997 |
| MSD+C+P | MSD | 0.003 |
|  | MSD+NPA | 0.000 |
|  | MSD+C+P+NPA | 0.002 |
| MSD+NPA | MSD | 0.001 |
|  | MSD+C+P | 0.000 |
|  | MSD+C+P+NPA | 0.002 |
| MSD+C+P+NPA | MSD | 0.997 |
|  | MSD+C+P | 0.002 |
|  | MSD+NPA | 0.002 |

**Fig. 3D-alkaline soil**

| I | J | p values |
| --- | --- | --- |
| MSD | MSD+C+P | 0.000 |
|  | MSD+NPA | 0.000 |
|  | MSD+C+P+NPA | 0.888 |
| MSD+C+P | MSD | 0.000 |
|  | MSD+NPA | 0.000 |
|  | MSD+C+P+NPA | 0.000 |
| MSD+NPA | MSD | 0.000 |
|  | MSD+C+P | 0.000 |
|  | MSD+C+P+NPA | 0.000 |
| MSD+C+P+NPA | MSD | 0.888 |
|  | MSD+C+P | 0.000 |
|  | MSD+NPA | 0.000 |

The exact *p* values in **Fig. 3** are 9.5×10^−5^ (C. culicis vs. △trpc), 8.8×10^−7^ (P. polymyxa vs. △ipdc) for **E** and **F**.

**Fig. 3G-acid soil**

| I | J | p values |
| --- | --- | --- |
| MSD | MSD+C | 0.032 |
|  | MSD+trpc | 1.000 |
|  | MSD+P | 0.020 |
|  | MSD+ipdc | 0.378 |
| MSD+C | MSD | 0.032 |
|  | MSD+trpc | 0.032 |
|  | MSD+P | 1.000 |
|  | MSD+ipdc | 0.000 |
| MSD+trpc | MSD | 1.000 |
|  | MSD+C | 0.032 |
|  | MSD+P | 0.200 |
|  | MSD+ipdc | 0.378 |
| MSD+P | MSD | 0.020 |
|  | MSD+C | 1.000 |
|  | MSD+trpc | 0.020 |
|  | MSD+ipdc | 0.000 |
| MSD+ipdc | MSD | 0.378 |
|  | MSD+C | 0.000 |
|  | MSD+trpc | 0.378 |
|  | MSD+P | 0.000 |

**Fig. 3G-alkaline soil**

| I | J | p values |
| --- | --- | --- |
| MSD | MSD+C | 0.000 |
|  | MSD+trpc | 0.955 |
|  | MSD+P | 0.000 |
|  | MSD+ipdc | 0.992 |
| MSD+C | MSD | 0.000 |
|  | MSD+trpc | 0.002 |
|  | MSD+P | 1.000 |
|  | MSD+ipdc | 0.001 |
| MSD+trpc | MSD | 0.955 |
|  | MSD+C | 0.002 |
|  | MSD+P | 0.002 |
|  | MSD+ipdc | 0.999 |
| MSD+P | MSD | 0.000 |
|  | MSD+C | 1.000 |
|  | MSD+trpc | 0.002 |
|  | MSD+ipdc | 0.001 |
| MSD+ipdc | MSD | 0.992 |
|  | MSD+C | 0.001 |
|  | MSD+trpc | 0.999 |
|  | MSD+P | 0.001 |

**Fig. 3H-acid soil**

| I | J | p values |
| --- | --- | --- |
| MSD | MSD+C | 0.012 |
|  | MSD+trpc | 1.000 |
|  | MSD+P | 0.011 |
|  | MSD+ipdc | 0.510 |
| MSD+C | MSD | 0.012 |
|  | MSD+trpc | 0.016 |
|  | MSD+P | 1.000 |
|  | MSD+ipdc | 0.000 |
| MSD+trpc | MSD | 1.000 |
|  | MSD+C | 0.016 |
|  | MSD+P | 0.014 |
|  | MSD+ipdc | 0.447 |
| MSD+P | MSD | 0.011 |
|  | MSD+C | 1.000 |
|  | MSD+trpc | 0.014 |
|  | MSD+ipdc | 0.000 |
| MSD+ipdc | MSD | 0.510 |
|  | MSD+C | 0.000 |
|  | MSD+trpc | 0.447 |
|  | MSD+P | 0.000 |

**Fig. 3H-alkaline soil**

| I | J | p values |
| --- | --- | --- |
| MSD | MSD+C | 0.001 |
|  | MSD+trpc | 0.952 |
|  | MSD+P | 0.000 |
|  | MSD+ipdc | 0.999 |
| MSD+C | MSD | 0.001 |
|  | MSD+trpc | 0.004 |
|  | MSD+P | 0.998 |
|  | MSD+ipdc | 0.000 |
| MSD+trpc | MSD | 0.952 |
|  | MSD+C | 0.004 |
|  | MSD+P | 0.002 |
|  | MSD+ipdc | 0.875 |
| MSD+P | MSD | 0.000 |
|  | MSD+C | 0.998 |
|  | MSD+trpc | 0.002 |
|  | MSD+ipdc | 0.000 |
| MSD+ipdc | MSD | 0.999 |
|  | MSD+C | 0.000 |
|  | MSD+trpc | 0.875 |
|  | MSD+P | 0.000 |

4. The exact *p* values in **FIG.4** are 0.042 (control vs. +C+P) of WT, 0.014 (control vs. +C+P) of nrh for **A**; 0.0006 (control vs. +C+P) of WT, 0.006 (control vs. +C+P) of nrh for **B**; 0.0068 (control vs. +C+P) of WT, 0.004 (control vs. +C+P) of nrh for **C**; 0.0026 (control vs. +C+P) of WT, 0.045 (control vs. +C+P) of nrh for **D**.

5. The exact *p* values in **Fig. S1** are 9.2×10^−10^ (WT-AC vs. WT-AL), 0.012 (nrh-AC vs. nrh-AL) for **B**.

6. The exact *p* values in **Fig. S8** are 9.2×10^−6^ (Control vs. +C+P) of WT, 0.0016 (Control vs. +C+P) of nrh for **A**; 2.4×10^−6^ (Control vs. +C+P) of WT, 0.0061 (Control vs. +C+P) of nrh for **B**; *p* < 0.0001 for **C** and **D**.

7. **Table S3-Sanming**

|  |  | p values | | | | | | | |
| --- | --- | --- | --- | --- | --- | --- | --- | --- | --- |
| Sanming |  | Plant height | Spike length | Grain number spike^-1^ | Filled grain rate | Thousand kernels weight | Grain length | Grain width | Harvest index |
| WT | WT+C+P | 0.451 | 0.972 | 0.983 | 0.604 | 1.000 | 0.990 | 0.978 | 0.457 |
|  | nrh | 0.675 | 0.613 | 1.000 | 1.000 | 0.140 | 0.990 | 0.993 | 0.859 |
|  | nrh+C+P | 0.724 | 1.000 | 0.968 | 0.347 | 0.786 | 0.070 | 0.911 | 0.587 |
| WT+C+P | WT | 0.451 | 0.972 | 0.983 | 0.604 | 1.000 | 0.990 | 0.978 | 0.457 |
|  | nrh | 0.981 | 0.854 | 0.968 | 0.646 | 0.157 | 1.000 | 0.911 | 0.135 |
|  | nrh+C+P | 0.967 | 0.956 | 0.845 | 0.968 | 0.818 | 0.124 | 0.719 | 0.052 |
| nrh | WT | 0.675 | 0.613 | 1.000 | 1.000 | 0.140 | 0.990 | 0.993 | 0.859 |
|  | WT+C+P | 0.981 | 0.854 | 0.968 | 0.646 | 0.157 | 1.000 | 0.911 | 0.135 |
|  | nrh+C+P | 1.000 | 0.569 | 0.983 | 0.382 | 0.557 | 0.124 | 0.978 | 0.961 |
| nrh+C+P | WT | 0.724 | 1.000 | 0.968 | 0.347 | 0.786 | 0.070 | 0.911 | 0.587 |
|  | WT+C+P | 0.967 | 0.956 | 0.845 | 0.968 | 0.818 | 0.124 | 0.719 | 0.052 |
|  | nrh | 1.000 | 0.569 | 0.983 | 0.382 | 0.557 | 0.124 | 0.978 | 0.961 |

**Table S3-Yangzhou**

|  |  | p values | | | | | | | |
| --- | --- | --- | --- | --- | --- | --- | --- | --- | --- |
| Yangzhou |  | Plant height | Spike length | Grain number spike^-1^ | Filled grain rate | Thousand kernels weight | Grain length | Grain width | Harvest index |
| WT | WT+C+P | 0.891 | 1.000 | 0.843 | 0.504 | 0.294 | 0.923 | 0.759 | 0.419 |
|  | nrh | 0.970 | 0.854 | 1.000 | 0.894 | 0.300 | 0.996 | 0.996 | 0.995 |
|  | nrh+C+P | 0.998 | 0.998 | 0.946 | 0.135 | 0.677 | 0.171 | 0.565 | 0.489 |
| WT+C+P | WT | 0.891 | 1.000 | 0.843 | 0.504 | 0.294 | 0.923 | 0.759 | 0.419 |
|  | nrh | 0.658 | 0.854 | 0.883 | 0.891 | 1.000 | 0.830 | 0.868 | 0.300 |
|  | nrh+C+P | 0.949 | 0.998 | 0.530 | 0.821 | 0.899 | 0.439 | 0.987 | 0.999 |
| nrh | WT | 0.970 | 0.854 | 1.000 | 0.894 | 0.300 | 0.996 | 0.996 | 0.995 |
|  | WT+C+P | 0.658 | 0.854 | 0.883 | 0.891 | 1.000 | 0.830 | 0.868 | 0.300 |
|  | nrh+C+P | 0.923 | 0.927 | 0.918 | 0.412 | 0.904 | 0.113 | 0.696 | 0.359 |
| nrh+C+P | WT | 0.998 | 0.998 | 0.946 | 0.135 | 0.677 | 0.171 | 0.565 | 0.489 |
|  | WT+C+P | 0.949 | 0.998 | 0.530 | 0.821 | 0.899 | 0.439 | 0.987 | 0.999 |
|  | nrh | 0.923 | 0.927 | 0.918 | 0.412 | 0.904 | 0.113 | 0.696 | 0.359 |
